# Priority effects inhibit the repeated evolution of phototrophy

**DOI:** 10.1038/s44260-026-00069-z

**Published:** 2026-02-02

**Authors:** Anthony J. Burnetti, James T. Stroud, William C. Ratcliff

**Affiliations:** School of Biological Sciences, Georgia Institute of Technology, Atlanta, GA USA

**Keywords:** Ecology, Ecology, Evolution, Plant sciences

## Abstract

The emergence of phototrophy is one of the most significant innovations in the history of life, vastly increasing available metabolic energy. Phototrophy is, however, known to have arisen only twice. This raises a curious question: if phototrophy was accessible enough to evolve twice, why has it never arisen again despite billions of years of subsequent evolution? Through physiological modeling, we demonstrate that chlorophototrophy and retinalophototrophy together saturate the bioenergetic landscape available to light-harvesting systems. They represent opposite solutions to key biophysical trade-offs: maximizing efficiency per photon versus maximizing metabolic flux, specialization versus versatility, and sophistication versus simplicity. Together they create an evolutionary priority effect, blocking any newly-arising phototrophic system from succeeding. By revealing the basis of this competitive exclusion, our work sheds light on a general principle - that early innovations can saturate ecological space such that they constrain future evolutionary possibilities, making apparently ‘easy’ innovations appear as rare events.

## Introduction

Major evolutionary innovations, from multicellularity to powered flight have repeatedly transformed life on Earth. Understanding how and why these novelties emerge and persist is therefore central to explaining life’s history. These innovations, however, often follow two very different evolutionary paths: while some innovations, like multicellularity^1^ or each of the varied forms of chromosomal sex determination^2^, have evolved dozens of times across diverse lineages, others remain restricted to just one or a few groups. These can even appear as true evolutionary singularities, such as the origin of life or eukaryogenesis. It has been argued that some of these singularities could represent rare restrictive bottlenecks that we only observe due to anthropic selection effects^3^, deterministic necessities which could only have ever occurred one way, or attrition of multiple origins by extinction over time^4^. One insufficiently explored potential explanation for this pattern, however, is priority effects: When an innovation first appears, the pioneering lineage may saturate the available ecological niche space, creating competitive barriers that prevent the same innovation from being successful if it evolves de novo in other groups^5,6^. This notion represents a fundamental connection between ecology and macroevolution at the largest scales – local interactions between organisms may have far-reaching evolutionary consequences at the level of fundamental biosphere-scale innovations^7,8^. Charles Darwin himself indirectly invoked this idea in a letter, speculating on why multiple separate origins of life are not ongoing today: “at the present day such matter would be instantly devoured or absorbed, which would not have been the case before living creatures were formed”^9^.

Despite the recognized importance of such niche incumbency in shaping macroevolutionary patterns^10–12^, the mechanistic basis of priority effects often remains poorly understood^5^. Here, we study how priority effects may manifest in major evolutionary innovations by studying the physiological bioenergetics of phototrophy. Phototrophy, the ability to use light for metabolic energy, is a major biological innovation that led to an explosion of biomass and biodiversity. However, despite first appearing at least 3.5Ga ago^13–15^ and being responsible for the vast majority of biomass on Earth^16,17^, it is unclear why phototrophy has only ever evolved independently in two forms: chlorophototrophy and retinalophototrophy. This low, but nonzero, diversity of phototrophic origins represents a unique opportunity for study—while not a *common* evolutionary innovation with sufficient barriers to its evolution to have only appeared twice, phototrophy nonetheless exhibits two separate origins that can be compared to determine the eco-evolutionary relationship between them. Could evolutionary priority effects explain their relationship, and what can we learn about the ecological interactions that engender such priority effects?

To address this question, we develop a mathematical analysis of energy transduction through these modern extant phototrophic metabolisms, and a biophysical model of the trade-offs inherent in their adaptation to different phototrophic ecological niches. We test the hypothesis that these two extant systems are optimized for different ecological conditions by their fundamental biophysical properties, and find that they indeed efficiently partition phototrophic niche space between themselves, limiting the opportunity for novel phototrophic lineages to establish themselves. This suggests that the ‘dual singularity’ of phototrophy exemplifies a fundamental principle: evolutionary innovations can be historically contingent with early-arising forms constraining the evolutionary possibilities available to later lineages. This further suggests that these major innovations, including but not limited to phototrophy, could be much simpler and more likely to evolve than their rarity would otherwise indicate with major implications for both the development of life on Earth and for the broader field of astrobiology.

**Fig. 1:**
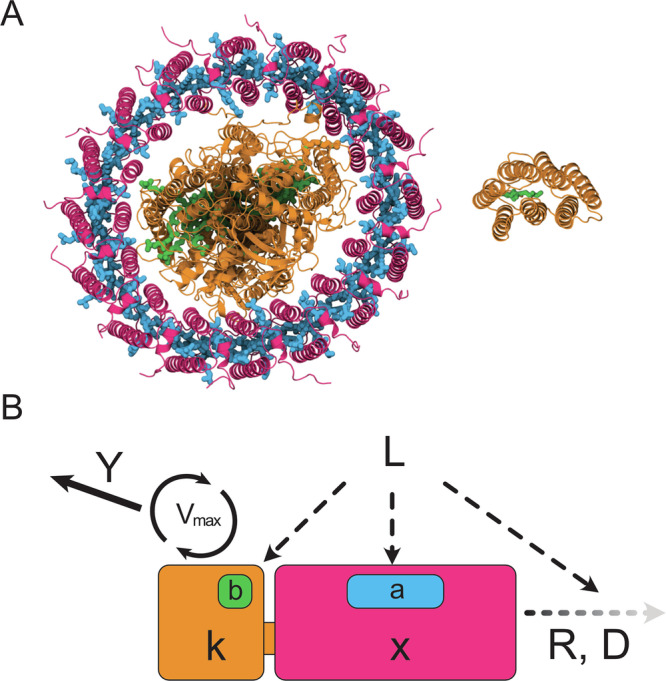
Divergent phototrophic machineries and their functional models. **A** Structural comparison of chlorophyll-based and retinal-based photosystems. Left: Type II reaction center with antenna complex from *Thermochromatium tepidum*^88^. The catalytic core (orange) contains bacteriochlorophyll pigments (green) that drive electron transport and carbon fixation, while antenna complexes (magenta) with additional pigments (blue) expand light capture. Right: Bacteriorhodopsin, a single transmembrane protein (orange) that pumps one proton per photon using a retinal chromophore (green)^94^. **B** Biophysical model parameters corresponding to structural components described above. Each system has an invariant catalytic core (orange, mass *k*) with maximum reaction rate *V*_*max*_ and proton yield *Y* per cycle. Core light absorption is *b*, while antenna mass *x* (magenta) provides additional absorption capacity *a* per unit mass. Environmental parameters include incident light intensity *L*, protein recycling rate *R*, and photodegradation constant *D*. Protein structures in (**A**) were visualized using Protein Imager^95^.

Chlorophototrophic and retinalophototrophic pathways originated independently early in the history of life on Earth. They are highly divergent in their structures, compositions, mechanisms of action, and the forms of chemical energy they make available to cellular metabolism (Fig. 1A). Before describing our computational approach for investigating their evolution and interactions, we provide a brief overview of these two phototrophic systems.

Chlorophototrophy drives both energy metabolism and redox chemistry using chlorophyll and bacteriochlorophyll photochemistry, with these tetrapyrrole pigments being evolutionarily derived from heme^18,19^. Found in bacteria and in eukaryotic algae in which cyanobacteria have been taken up as chloroplasts, chlorophototrophy is responsible for the vast majority of primary production on the planet^16,17^. The functional unit of chlorophototrophy is the photochemical reaction center (RC; Fig. 1A), which are large transmembrane protein complexes agreed to be descended from an ancestral homodimer^15,20^. The deepest split in their phylogeny is between type I RCs that use light energy to reduce ferredoxin using electrons from cytochromes or plastocyanin, and type II RCs that reduce quinones with electrons from cytochromes or water. The diversity of reaction centers, the electron transport chains they are integrated with, and the metabolisms they contribute to are profound^21^, with multiple anoxygenic systems coexisting on Earth with oxygenic photosynthesis and a surprising diversity of metabolic capacities found even within just the oxygen-producing cyanobacteria^22^. The details of the relationships between these forms is a matter of some debate, but largely not relevant to our discussion which rests on common aspects shared by all of them. All RCs contain a minimum of eight conserved central organic pigment and redox cofactor molecules^23,24^, carefully arranged in space to allow rapid charge separation and pointing to the form of their common ancestor. In addition to their stereotypical tetrapyrrole pigments and quinone cofactors, all modern reaction centers contain iron in the form of iron-sulfur clusters, single coordinated iron atoms, or hemes bound to cytochromes^25–28^ and are functionally integrated into operational electron transport chains.

All chlorophototrophic reaction centers are united in generating reducing power via chlorophyll and bacteriochlorophyll photochemistry. This reducing power can then be used for energy metabolism by allowing reduced electrons to flow through a modified electron transport chain or used as a reductant for carbon/nitrogen fixation. In the case of energy metabolism, protons are pumped across the active membrane much as in a respiratory electron transport chain. In the case of carbon fixation, a reducing equivalent is conveyed to NADPH while the reaction center is “reset” by pulling an electron from a variety of environmental electron sources (such as dissolved Fe^2+^ in photoferrotrophs, H_2_S in green sulfur bacteria, or water in the case of oxygenic photosynthesis). Absorption of a single photon typically pumps two to four protons across the membrane^29–31^. All reaction centers couple a structurally conserved central dimeric core of at least 150 kDa^23^, coupled physically to additional ‘core antenna’ complexes of a hundred kDa or more which increase the absorption cross section per functional unit by funneling light into the reaction center from additional pigments via Förster resonance transfer. These in turn may be coupled to a diverse variety of additional antenna complexes which vary significantly from lineage to lineage^32^.

Retinalophototrophy, in contrast, is mediated by a single simple 26–28 kDa transmembrane protein, known as a microbial or “type-1” rhodopsin^33–35^ (Fig. 1A). It is covalently bound to a single pigment molecule known as retinal, derived from the splitting of a carotenoid via a dioxygenase^36^ – it is notable that in both the case of retinal and chlorophyll, the pigment synthesis pathway is derived from the synthesis of cellular components with non-phototrophic roles. In a few cases, such as the xanthorhodopsins, a single additional carotenoid pigment molecule is bound to the exterior of the protein and functions as a miniature integral antenna^37^, but rhodopsins are not known to be associated with accessory antenna proteins.

Rather than participating in redox chemistry, rhodopsins directly pump protons across the cell membrane driven by light-induced isomerization of the retinal pigment molecule. Thus unlike chlorophototrophic systems they involve only organic pigments that operate directly upon ion flux, with no need for metal ion cofactors and no ability to produce the reducing power needed to directly drive a dedicated carbon fixation pathway^38^. Rhodopsins are not known to be used for autotrophic fixation of CO_2_ into biomass, only accelerating anaplerotic reactions during heterotrophic growth^39^ in nature, likely due to them conserving relatively little energy per photon into membrane potential^31^ and raising the membrane voltage to a lower voltage than electron transport chains do^40^. This prevents their use to drive the inefficient reverse electron flow backwards through an electron transport chain necessary for true autotrophic carbon fixation in the absence of direct NADPH production by chlorophototrophy^41^. However, while these two phototrophic systems are quite different, the total energy transduced by them in the biosphere is similar. The quantity of light absorbed by retinalophototrophs in the ocean is thought to be at least as large as that absorbed by chlorophototrophs^42^.

## Results

### Extant chlorophototrophs and retinalophototrophs

**Fig. 2:**
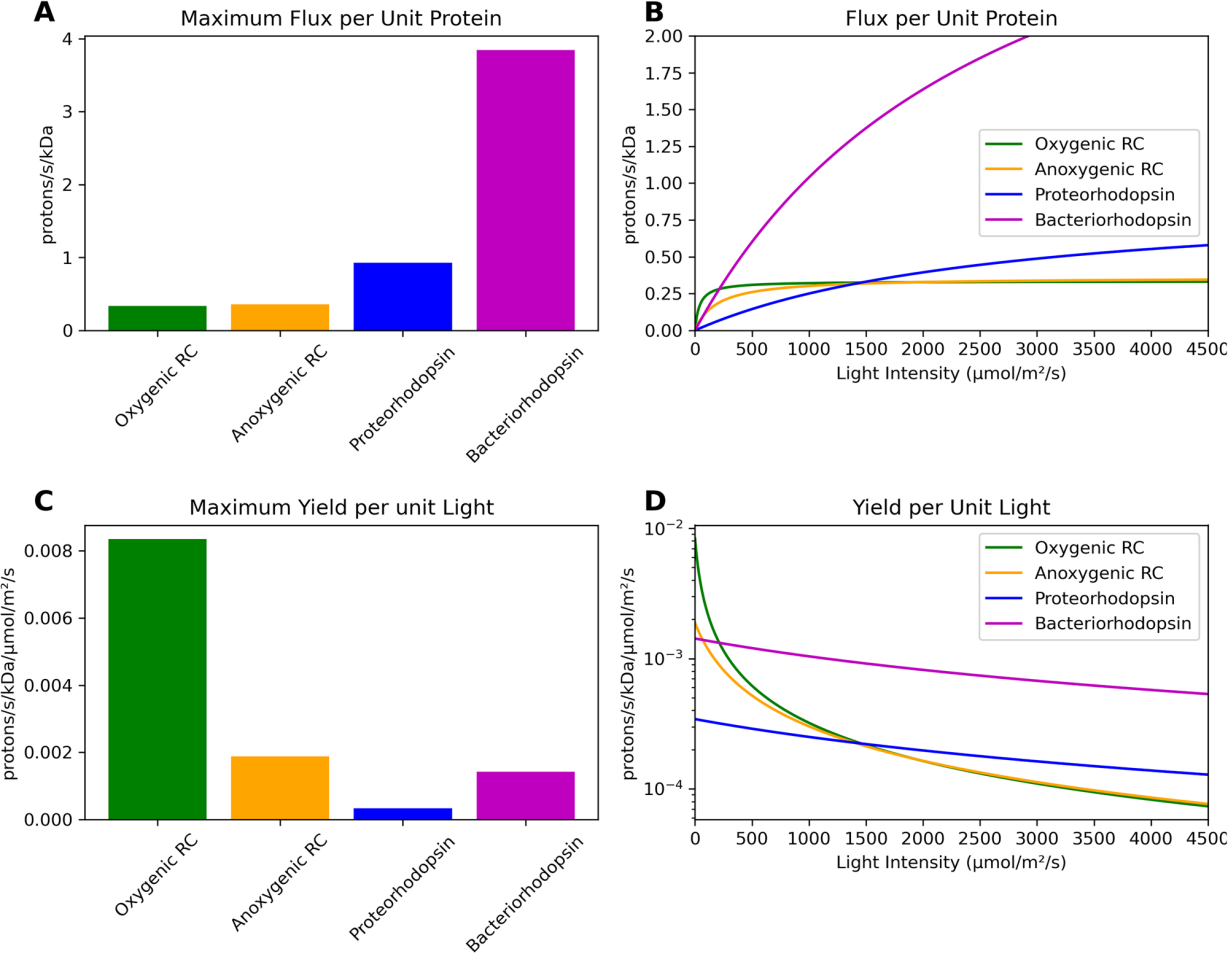
Physiological comparison between modern chlorophototrophs and retinalophototrophs reveals fundamental differences in how these systems harness light energy. When examining maximum proton flux at saturating light levels (**A**), retinal-based systems like proteorhodopsin and bacteriorhodopsin dramatically outperform their chlorophyll-based counterparts, achieving much higher specific fluxes per unit of protein mass than either oxygenic or anoxygenic reaction centers. This advantage shifts dramatically across different light conditions (**B**), where chlorophyll systems demonstrate superior performance at low light intensities but quickly reach saturation, while retinal systems continue to increase their flux as light intensities rise, ultimately achieving higher maximum rates. The underlying reason for this trade-off becomes clear when examining light-use efficiency (**C**), which shows that chlorophyll systems extract far more energy from each individual photon than retinal systems, with oxygenic reaction centers showing particularly impressive yields per unit of incident light. This pattern persists across the full spectrum of light intensities (**D**), consistently demonstrating that chlorophototrophs have evolved to maximize energy extraction under scarce light conditions, while retinalophototrophs have specialized for high-flux energy generation when light is abundant.

We first examined the physiological trade-offs embodied in chlorophototrophic and retinalophototrophic machinery using data derived from physiological studies of extant taxa based on a literature review^23,27,34,40,43–52^, calculating their machinery’s energy flux per kDa and energy yield per unit light intensity at different light intensities and in the limits of bright and dim light (see Supplement S1 and Table S1). Despite possessing both a faster photocycle and higher energy yield per photon absorbed, chlorophototrophic machinery has a substantially lower maximum specific energy flux per unit mass than microbial rhodopsins (Fig. 2A; Supplementary Table S1). This is principally because of the much larger mass of chlorophototrophic reaction centers and their associated antennas.

Upon analysis of the function of RCs and rhodopsins at a variety of light intensities, we find that the small protein mass and presence of only a single retinal pigment in a rhodopsin ensures a small light absorption cross section which requires intense ambient light for the machinery to be used effectively. The larger size of a reaction center comes with more pigment molecules and a larger light absorption cross section which allows a higher specific energy flux at low light intensities, but this rapidly saturates and is overtaken by rhodopsins at high light intensities (Fig. 2B). *A clear trade-off between these two pathways exists*: while rhodopsins are capable of much higher maximum energy fluxes per unit protein infrastructure at high light levels, RCs are capable of much higher maximum energy yields per unit incident light than retinalophototrophs at low light levels (Fig. 2C, D). This is likely related to a deep, fundamental thermodynamic trade-off between the yield and flux of a biochemical reaction – if more energy is captured per reaction cycle, the thermodynamic driving force of the reaction is decreased and the reaction rate along with it^53^. Similar trade-offs exist in other metabolic pathways, such as the difference between the EMP (high yield) and ED (high flux) glycolytic pathways^54^.

### Theoretical Trade-Offs

Moving on from these well-studied modern phototrophic organisms, we next examined the fundamental theoretical physiological capabilities of chlorophototrophy and retinalophototrophy using an integrated physiological model of phototrophic energy transduction (Fig. 1B, Supplement S1, Table S2). This model takes into account the properties of the conserved catalytic cores at the heart of rhodopsins and reaction centers, variable quantities of associated antenna pigments increasing the light-gathering cross section of the machinery, and both the photodegradation and growth/recycling-mediated dilution of the machinery with time. Light levels were allowed to vary between 0.01 to 4000 micromoles of photons per square meter per second in logarithmic intervals, and the optimal quantity of antenna pigment per catalytic core to optimize specific energy flux of the protein machinery at each light intensity was calculated. The properties of each of these optimal systems was analyzed in terms of specific energy flux per unit protein (protons pumped per second per kDa of protein) and yield per unit incident light (protons pumped per second per kDa of protein per unit of incoming light intensity; see “Materials and Methods”).

**Fig. 3:**
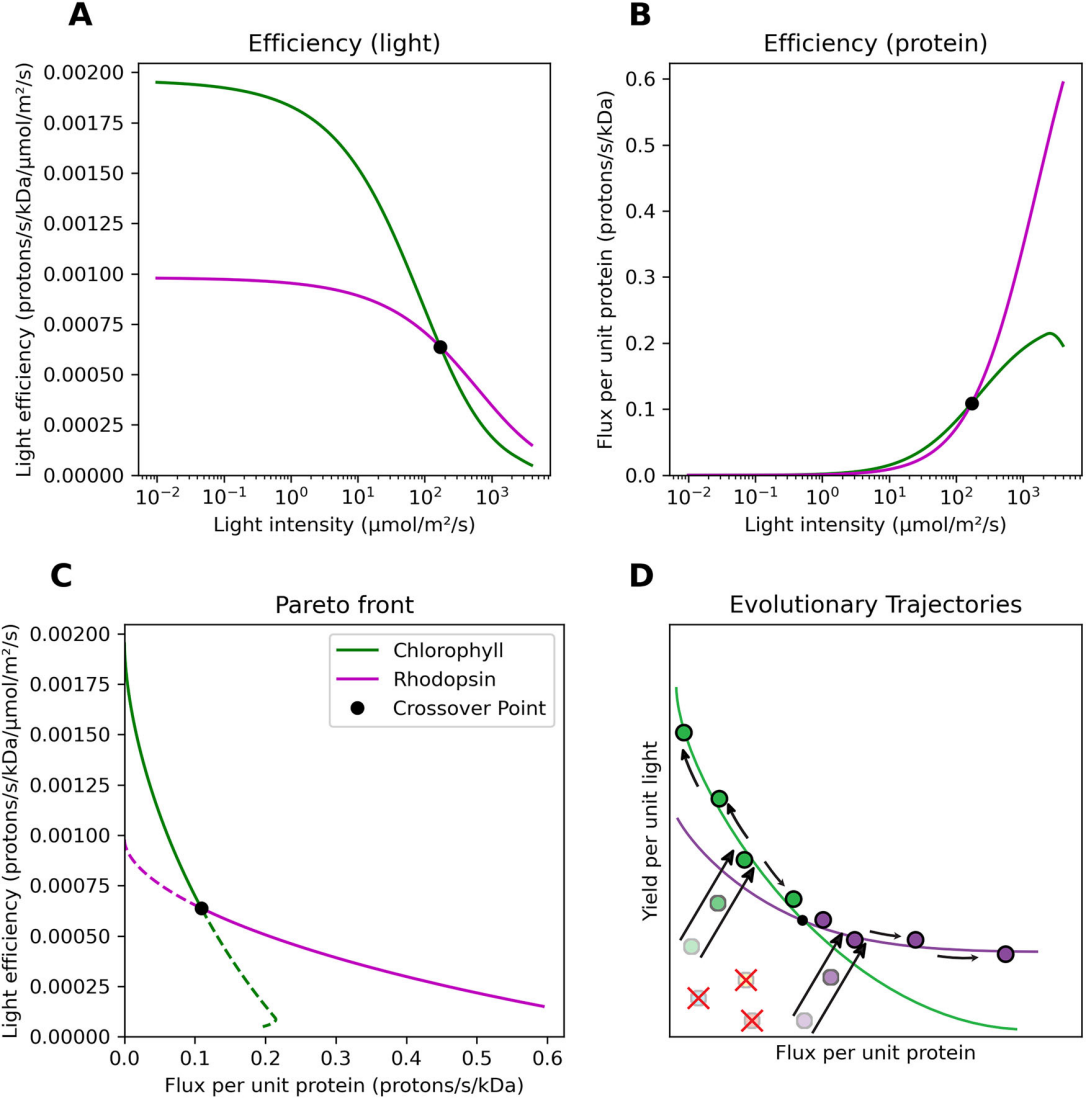
Fundamental trade-offs in phototrophic machinery dictate ecological interactions and evolutionary trajectories. **A** Optimal chlorophototrophs maximize light efficiency in low light while optimal retinalphototrophs excel in high light. **B** This specialization persists when examining energy flux per unit protein, with each system showing superior flux in the same light conditions. **C** Trade-off between light efficiency and protein efficiency across all light intensities. Each system creates a Pareto front showing the maximum achievable efficiency combinations. The crossing curves demonstrate that chlorophototrophs dominate in some conditions (solid green) while retinalphototrophs dominate in others (solid purple), with non-competitive regions shown as dotted lines. **D** Evolutionary model showing priority effects in photosynthetic diversification. Modern chlorophototrophs (dark green) and retinalphototrophs (dark purple) occupy optimal positions along their respective Pareto fronts. Early, less efficient ancestors (light circles) rapidly evolved toward these optimal curves and diverged into distinct ecologies (arrows). These established systems now prevent evolution of competing pathways (red crosses) because new systems would be initially less efficient and quickly outcompeted.

Yield per unit incident light falls nonlinearly as light intensity increases (Fig. 3A) due to both saturation of the central catalytic core and drastic decreases in optimal antenna quantity (see Figs. S2 and S3). Efficiency per unit protein machinery, conversely, rises nonlinearly as light intensity increases (Fig. 3B) due to increased utilization of the catalytic core and a smaller antenna mass, with chlorophototrophs exhibiting a slightly decreased efficiency at the highest light intensities due to photodegradation. Our analysis identifies a crossover point at ca. 186 micromoles of photons per square meter per second (Fig. 3A, B). Above this light intensity, both the energy flux per unit infrastructure and energy yield per unit incident light are superior for retinalophototrophy. Below this light intensity, chlorophototrophy instead dominates for both metrics. Light intensity thus determines which phototrophic system will be more efficient, with two defined “niches”—a low light niche with high yield per unit light, and a high light niche with high flux per unit infrastructure.

Our model recapitulates several key aspects of phototrophic metabolism observed in nature. It indicates a theoretical minimum optimal antenna mass of ~95 kDa for optimal chlorophototrophs at the crossover point light level (Fig. S2), with antenna mass being able to increase without limit at low light levels. The lower chlorophototrophic antenna limit is within ~20% of the mass of the smallest intrinsic antennas observed in nature in type I reaction centers, while other chlorophototrophs can indeed exhibit extremely large antenna pigments up to and including ‘chlorosome’ antennas the size of organelles^55^. While we calculate a maximum antenna size of 61 kDa for theoretical antennas associated with rhodopsins at the crossover point, this predicted mass is below the mass of any observed antenna complex and rapidly falls to negligible optimal mass as light intensity increases towards that of full sunlight (see Fig. S2). In addition to this, the crossover point at which higher light intensities are dominated by retinalophototrophy corresponds closely with the empirically observed light intensities above which retinal is over-represented in the ocean and chlorophyll is under-represented^42^ (see File S1 and Fig. S5). Our simple model thus recapitulates key physiological trade-offs underlying variation in organisms filling phototrophic niche space, able to correctly predict the environmental conditions under which either chlorophototrophs or retinalophototrophs should have a physiological and ecological advantage.

## Discussion

Phototrophic organisms face a fundamental trade-off between efficiency per unit light and flux per unit infrastructure. The two phototrophic metabolisms that have evolved on Earth have each optimized a different side of this trade-off: chlorophototrophy requires a significant fraction of the proteome to be invested in sophisticated protein machinery which results in a lower energy flux per unit infrastructure but high yield per unit light, while retinalophototrophy results in a high energy flux per unit of simple infrastructure but lower yield per unit light.

As a pathway with high yield per unit light, chlorophototrophy allows the capture of dilute light resources for obligate phototrophs, even in difficult conditions, at the expense of high protein expression and specialization of more of the proteome. As a pathway with a higher flux per unit protein, retinalophototrophy can instead provide a benefit in high light with less total expression of a simple protein, allowing it to be used as a backstop to prevent starvation and increase metabolic flexibility and biomass yield of otherwise heterotrophic organisms without specialization. These two forms of photosystems effectively partition the metabolic niche space available to phototrophs, both in terms of resource availability and in terms of the degree of phototrophic specialization.

By graphing flux per unit protein versus yield per unit resource of optimal phototrophic systems for every tested light intensity, we can create a diagram of phototrophic niche space. It becomes apparent that the optimal physiological machinery for a given environment lies along a Pareto front^56,57^ (Fig. 3C) with the position along this front dictated by light intensity. Evolution should push novel phototrophic lineages towards this front from any suboptimal starting point they originate from (Fig. 3D). Each phototrophic machinery type exhibits its own Pareto front with differing slopes; the point at which they cross indicates the light intensity separating niches in which one or the other is favored for driving phototrophic energy metabolism.

Why has neither phototrophic strategy completely dominated all available niche space? It would appear that their fundamental architectures are sufficiently different that they have deeply different advantages. It is illuminating that not only do these two systems coexist in Earth’s biosphere, in some cases they even coexist within the same organism—there are multiple known bacteria^58–60^ marine diatoms^61–63^ and phytoplankton^64,65^ which carry both chlorophototrophic and retinalophototrophic machinery. They are frequently differentially expressed in different environments, especially differential light intensity^58^ and iron availability^61^. Rather than any organism being able to optimize a single system for a wider range of environments, it is instead apparently preferable to include both systems to cover the full range when necessary.

Fundamental structural aspects of the catalytic cores of each phototrophic system appear to ensure that the extremes of the investment/resource efficiency trade-off are only accessible to one or the other. Microbial rhodopsins responsible for retinalophototrophy are simple, direct light-driven proton pumps driven by the physical isomerization of a single organic molecule. This limits the energy captured per photon absorbed to the work done on one proton as it is pushed across the membrane potential. Without any redox-active cofactors in its structure, which would operate through a fundamentally different mechanism, rhodopsin cannot be recruited to interact with the electron transport chains needed to pump multiple protons per photon or to engage in redox metabolism. Retinalophototrophy therefore appears to be incapable of evolving to pump more than one proton per photon and efficiently use available light resources, although its small mass means it enjoys a high maximum specific energy flux. Conversely, the chlorophototrophic reaction center depends on careful arrangement of multiple pigments and redox cofactors in space relative to each other to allow light-driven redox reactions that conserve more energy – a pair of tetrapyrrole pigments must be present for light-driven charge separation, and additional carefully arranged pigments and cofactors ensure that the separated charges are delocalized rapidly and remain apart and stable, without wastefully losing reducing power to recombination^66^. This more complex setup can conserve more energy but seemingly cannot be reduced below a relatively large minimum size in modern organisms, with all known examples massing at least 150 kDa and containing at least eight pigment and quinone molecules. This limits its maximum energy flux per unit mass, even as it enjoys a high efficiency per unit light captured due to conserving more energy per photon. Due to architectural and thermodynamic constraints, chlorophototrophy and retinalophototrophy coexist as energy metabolisms via divergent ecological and physiological trade-offs.

The observed flux versus yield trade-off is likely in part the result of the basic thermodynamic and kinetic limits of biochemistry. Reactions that conserve more energy generally run more slowly due to a smaller fraction of available free energy being dissipated^53^, or require larger more sophisticated catalysts^67^, in either case resulting in a lower flux per unit catalyst. This trade-off manifesting across ecological niches of differing resource availability has been observed in non-phototrophs as well. The difference between respiration and fermentation is an example – respiration can produce several times the ATP per unit substrate consumed while producing less than half the energy flux per unit protein mass, with bacteria preferring respiration at low resource availability and fermentation at high^68^. The two most common glycolytic pathways, the Etner-Doudoroff (ED) and Embden-Meyerhof-Parnas (EMP) pathway, share this relationship as well with the EMP pathway producing twice the ATP per unit carbohydrate consumed as the ED pathway, but requiring 5-fold more protein mass^54^. Much as in phototrophy, the lower-flux higher-yield EMP pathway is seen more frequently than the ED pathway in obligate anaerobes which must use glycolysis for energy and obtain higher yield from limited available substrate. This trade-off can have important effects on growth rates and ecology, with cells requiring less proteome to be devoted to high-flux metabolic machinery able to put more productive capacity towards growth rather than metabolism resulting in a series of widespread ecologically relevant ‘bacterial growth laws’^68–70^.

As chlorophototrophy and retinalophototrophy dominate the available phototrophic niche space, there exists little opportunity for the establishment of additional phototrophic forms de novo. All modern phototrophs will be distributed along the combined Pareto front^56,57^ (Fig. 3C); any newly-evolved inefficient phototrophic system will inevitably be strictly inferior to both extant established forms (Fig. 3D). As such, chloro- and retinalophototrophy together exhibit strong priority effects^6,11,71,72^—otherwise known as niche incumbency^10^—which limits the establishment and success of novel photosystems which use the same resources as the established systems^11,73^ even if such systems were simple to evolve. This suggests a fundamental continuity between local ecological interactions involved niche colonization and succession and patterns of evolutionary innovation at the largest of scales, as has been suggested by Baum et al.^74^, and implies that local ecological interactions can drastically shape the long-term history of major evolutionary innovations.

However, even if today these phototrophic systems can coexist due to their divergent trade-offs, why did whichever one that appeared first not completely suppress the origin of the second due to the phenomenon of niche incumbency? One would think that whichever one came first would have rapidly evolved and optimized to the point that an unsophisticated and inefficient newcomer would be suppressed, even if it were theoretically capable of evolving to surpass the incumbent in some environments given enough time. It is difficult to determine from the paleontological record whether chlorophototrophs or retinalophototrophs evolved first—being unable to generate large amounts of biomass retinalophototrophy alone is unlikely to be visible in the fossil record, and presumably-chlorophototrophic microbial mats are seen approximately as far back in the rock record as it is possible to look^75,76^. However, it is possible to deduce a likely sequence of evolution and likely reason for coexistence from physiological analyses.

While chlorophototrophs are able to use light energy for either energy metabolism or the autotrophic fixation of CO_2_ into biomass, retinalophototrophs are only capable of using light for energy. While rhodopsins accelerate anaplerotic reactions taking up CO_2_^39^, this is inherently a component of heterotrophy that balances redox state rather than allowing for true autotrophy^77^. Rhodopsins are chemically incapable of driving redox reactions directly and fail to push the membrane to a high enough potential to run electron transport chains in reverse^40^, a precondition for driving carbon fixation via reverse electron flow^41^. This means that there is, effectively, a third dimension to the ecological niche of phototrophy representing the ability to fix carbon. Assuming that the earliest chlorophototrophs engaged in redox chemistry, even a very slow and poorly adapted proto-chlorophototroph would be superior to a retinalophototroph in its ability to autotrophically fix carbon without geologically provisioned electron sources. This would allow a way around any energy-metabolism-based priority effect engendered by an established retinalophototroph via a novel carbon fixation method, and an opportunity to evolve until it too became well-optimized. The deep mechanistic difference between the two then explains their coexistence after this optimization as previously described as they each fill their own area of the energy transduction trade-off curve, unable to successfully compete on the basis of energy in all environments. Our model therefore implies that retinalophototrophy likely evolved before chlorophototrophy given that carbon fixation allows a proto-chlorophototroph to avoid evolutionary priority effects from retinalophototrophs, but not the reverse.

An earlier origin of retinalophototrophy would be consistent with the fact that chlorophototrophy is significantly more complex than retinalophototrophy, requiring intact electron transport chains to function rather than merely the presence of a bioenergetically active membrane (likely present in the last universal common ancestor itself^78^). It could also explain the fact that the absorption spectrum of basal rhodopsins in the middle of the visible spectrum seems to directly mirror the spectrum available from sunlight underwater^79^ while chlorophylls and bacteriochlorophylls absorb long and short wavelength light that is less abundant but does not overlap with the absorption spectrum of retinal^80^. This is not likely the result of trade-offs in yield per photon at different wavelengths, as both rhodopsins^35^ and reaction centers^81^ have roughly constant yields per photon regardless of wavelength and chlorophototrophs intercept both shorter and longer wavelengths. This may instead represent a “spectral” priority effect embedded in the relationship between the colors of the two photopigments, based entirely on earlier incumbent photopigments using more common wavelengths since they arrived first and an initially inefficient newcomer using more readily available frequencies not attenuated by the incumbent.

In some ways this idea of an early origin of retinalophototrophy followed by a later origin of chlorophototrophy resembles the “purple earth hypothesis”, arguing that the early Earth was dominated by retinal photopigments and that similar pigments could be an astrobiological signature of young biospheres^80^. However, the apparent inability of rhodopsins to drive autotrophic carbon fixation argues against such a period being significant – with rhodopsin only able to enhance heterotrophy, any rhodopsin-only biosphere would remain of miniscule size, with chlorophototrophy increasing the scale of the realizable biosphere by a factor of a million or more^16,17^. Furthermore, if the primary factor limiting the origin of novel phototrophic pathways is indeed priority effects rather than difficulty of their invention the time between the origin of these two pathways could be geologically brief, as could the time between the origin of life itself and their origins. Our priority effect model thus has specific paleontological and astrobiological implications. It predicts that the time required to realize a fully photosynthetic autotrophic biosphere on Earth, and potentially elsewhere in the universe, is possibly quite short, consistent with evidence for phototrophic carbon fixation deep in the rock record. It also suggests that any given biosphere should possess at most two developed phototrophic pathways – one if the first pathway is capable of both carbon fixation and energy conversion, and two if the first is capable of only energy conversion.

There has been great debate about why some major evolutionary innovations exist only as singularities, while others independently evolve multiple times^4,82–84^. Our results suggest that priority effects may play a pivotal role in suppressing the repeated evolution of phototrophy; a phenomenon which may extend to the evolutionary patterns of some other major innovations. The evolution of complex cellular architecture, for example, is considered to have occurred only once on Earth in the form of eukaryogenesis. However, it is unclear if that is because the evolutionary pathway is complex and contingent or because eukaryotes have competitively excluded any secondary origins of such cellular complexity. A similar argument applies to the origin of life itself—is this a difficult process, or did modern life simply rapidly advance to a level of sophistication that scavenged all resources that could otherwise go towards simple novel replicators or protocells? As evidence for priority effects appears replete across biological, spatial, and temporal scales^5^, it is possible that such niche incumbency could play a pivotal role in the macroevolutionary landscape of multiple major innovations.

Phototrophy is among the most important innovations in the history of life, fundamentally changing the biosphere. It is unique among major biological innovations in that it has evolved not once, and not many times, but exactly twice. Here, we show that the two origins of phototrophy are mechanistically and ecologically complementary, having partitioned phototrophic niche space along a set of trade-offs that prevent either mechanism from becoming dominant. Deep architectural limitations and functional trade-offs inherent to the evolution of metabolic pathways appear to have prevented either chlorophototrophs or retinalophototrophs from occupying all phototrophic niche space individually, creating the opportunity for their stable coexistence. The remarkable fact that phototrophy evolved just twice, producing two ecologically complementary forms, however, reveals how priority effects can shape major innovations. While each established system likely prevented new phototrophic pathways from emerging through competitive exclusion, their fundamental differences in design and function meant neither could eliminate the other, allowing both to persist throughout Earth’s history.

It is tempting to take evolutionary rarity as a sign of intrinsic difficulty. Yet this interpretation assumes that evolutionary innovations represent independent rolls of the dice, each with the same low probability of success. Our results challenge this view. Phototrophy may not be intrinsically difficult to evolve, as evidenced by its dual origins through fundamentally disparate routes early in the history of life. Instead, we argue that the rarity of phototrophic innovations reflects the power of evolutionary priority effects: once chlorophototrophy and retinalophototrophy saturated the available metabolic niche space, they created insurmountable competitive barriers for any subsequent phototrophic systems, regardless of how readily such systems might have evolved in their absence. This perspective has profound implications for understanding other major transitions in life’s history, both on Earth and in other potential biospheres. The singularity of events like abiogenesis or eukaryogenesis may need not reflect vanishingly small probabilities or extraordinary confluences of unlikely circumstances. Instead, these sorts of innovations may have evolved with relatively high probability given appropriate conditions, but their first appearance triggered feedback processes that fundamentally altered the selective landscape, preventing future parallel evolution and making them appear to a first inspection more contingent than they actually are. A trait that evolves easily can still arise only once if its emergence forecloses the ecological opportunities necessary for its repeated evolution. In this light, life’s apparent evolutionary singularities may be less contingent than they appear, their rarity stemming not from intrinsic difficulty but from the consequences of priority effects operating across billions of years of Earth history.

## Methods

### Calculations and equations

We first calculated an effective specific energy flux per unit protein investment of different phototrophic systems based on a literature review of vital parameters for anoxygenic chlorophototrophic RCs, oxygenic RCs, and two different microbial rhodopsins (proteorhodopsin and bacteriorhodopsin)^23,24,27,34,40,43–52,85–90^. Vital parameters included total protein mass per functional unit *M*_*total*_ in kDa, the maximum rate *R*_*max*_ in cycles per second, protons pumped per cycle *N*_*p*_, and the light level at which absorption is at half-maximum *K*_*m*_, in units of micromoles of photons per square meter per second. Using Eq. 1 we calculated the maximum flux per unit protein at saturating light levels *V*_*max*_ (protons per second per kDa) (See Supplementary Tables S3 and S4).

1 $${V}_{\max }={N}_{p}\cdot {R}_{\max }/{M}_{{total}}$$

We extended this analysis from the maximal energy flux per unit mass to the flux per unit mass at differing light levels based on the absorption cross section and maximum photocycle rate *V*_*max*_ of different phototrophic machineries (see Supplementary File S1 and Supplementary Eq. S2 for a detailed description). We treated light absorption and conversion as a Michaelis-Menten process resulting in Eq. 2, describing the energy flux per unit protein *F*_*P*_ at a given light intensity *L* (see Supplementary Table S5).

2 $${F}_{P}=\frac{{V}_{\max }\cdot L}{{K}_{m}+L}$$

By dividing the function of the return per unit investment of each phototrophic system by the level of ambient light, we produced Eq. 3 describing the efficiency per unit ambient light *F*_*L*_, in units of protons pumped per kDa per second per micromole of photons per square meter per second and Eq. 4 describing the maximum energy flux per kDa per unit ambient light *Y*_*max*_ (Fig. 3C, D).

3 $${F}_{L}=\frac{{V}_{\max }}{{K}_{m}+L}$$

4 $${Y}_{\max }={V}_{\max }/{K}_{m}$$

In order to examine the theoretical capabilities of retinalophototrophs and chlorophototrophs, and the implications of the trade-offs embodied in either phototrophic system on organismal physiology, evolutionary history, and ecological interactions, we constructed an analytical model of an arbitrary phototrophic system and its photodegradation, recycling, and growth-based dilution^89–92^ (Fig. 1B). This model consists of the central catalytic core of the rhodopsin or reaction center with a constant yield, maximum reaction velocity, and absorption cross section. It is parameterized in terms of yield per cycle *Y* (protons per cycle), maximum rate *V*_*max*_ (photocycles per second), mass of catalytic core *k* (kDa), absorption cross section per catalytic core *b* (Å^2^), mass of antenna complexes *x* (kDa), absorption cross section per unit mass of antenna *a* (Å^2^ kDa^-1^) photodegradation constant *D* (photon^-1^), and recycling/dilution rate *R* (s^−1^).

The mass, optical properties, and energy-transduction properties of a chlorophototrophic catalytic core were imputed by examining an anoxygenic bacterial type II reaction center from *Thermochromatium tepidum*^23,24,88^ and adding up the absorption cross sections multiplied by the quantum efficiencies of their pigments^85–87^. The properties of microbial rhodopsins were imputed from bacteriorhodopsin^89,90,92^. The catalytic core is paired with antenna complexes of variable size, whose absorption cross section per unit mass was imputed by examination of the LH2 antenna of a purple nonsulfur bacterium^47,52,87^. Both chlorophototrophic reaction centers and rhodopsins were allowed to be paired or not paired with antenna complexes so as to not limit the model to configurations seen today, where only chlorophototrophs have antenna complexes larger than single carotenoid molecules, which could conceivably be due to historical contingency.

Our model follows Michaelis–Menten kinetics with regards to light absorption by its pigment cross-section and its conversion by the catalytic core—see Supplementary Eqs. S5–S10. Photodegradation properties and methods are taken from Han^93^ and Faizi et al.^91^ in which photodegradation is proportional to the rate of photon absorption by excited phototrophic machinery. We allowed both the catalytic core and antenna pigments to be subject to photodegradation. By combining the rate of photodegradation with a rate of dilution of degraded protein by growth and recycling, we derive the fraction of functional protein performing phototrophic energy conversion—see Supplementary Eqs. S11–S14. By applying this combined photodegradation and dilution correction to the flux of energy through the catalytic core (Eqs. 2 and 3), we arrive at Eq. 5 and Eq. 6 implemented in or model describing the flux per unit protein *F*_*P*_ (protons per kDa per second) and the yield per unit incident light *F*_*L*_ (protons per kDa per second per micromole of photons per square meter per second) of a phototrophic system.

5 $${F}_{P}=\frac{Y\cdot L\cdot {V}_{\max }\cdot ({ax}+b)}{{\left(k+x\right)\cdot (V}_{\max }+L\cdot ({ax}+b))}\cdot \frac{R}{R+\frac{{D\cdot L}^{2}\cdot {({ax}+b)}^{2}}{{V}_{\max }+L\cdot ({ax}+b)}}$$

6 $${F}_{L}=\frac{Y\cdot {V}_{\max }\cdot ({ax}+b)}{{\left(k+x\right)\cdot (V}_{\max }+L\cdot ({ax}+b))}\cdot \frac{R}{R+\frac{{D\cdot L}^{2}\cdot {({ax}+b)}^{2}}{{V}_{\max }+L\cdot ({ax}+b)}}$$

All parameters were constrained by review of previous literature except for the rate of recycling and dilution of phototrophic machinery by cell growth and division (R) which is highly dynamic depending on cellular doubling time and metabolic state. We took this value to be 0.1 h^−1^ in this analysis, corresponding to a protein half-life or cellular doubling time of 6.93 h, as this growth rate is comparable to those observed for rapidly growing chlorophototrophic algae and close to the upper growth rates modeled in Faizi et al.^91^. Varying this recycling and dilution timescale did not qualitatively impact our results—see Supplementary Fig. S4 for sensitivity analysis. See Supplementary Table S7 for all variables from the literature used in this theoretical analysis.

### Numerical simulations and analysis

We implemented models of extant phototrophic systems (Eqs. 1–4) and theoretical phototrophic systems (Eqs. 5–6) numerically in Python. For purposes of numerical calculation of optimal architectures and performances, we varied light intensity *L* the model was exposed to from 0.01 to 4000 micromoles of photons per square meter per second in 1000 logarithmically spaced intervals (the upper range is similar to the radiation intensity of full sunlight at the equator). At every light intensity, the optimal antenna mass *x* must be determined for chlorophototrophic machinery and for retinalophototrophic machinery. We maximized calculated energy flux per unit mass, as this optimization likely maximizes growth rate and thus fitness^68–70^. As solving the optimum antenna mass *x* for maximizing *F*_*P*_ and *F*_*L*_ was analytically intractable, we numerically approximated it to the nearest 0.1 kDa at every tested light intensity, creating a table of optimal antenna masses *x*_*opt*_ for each light intensity for both sets of machinery.

All previously described variables were calculated at each light intensity for chlorophototrophs and retinalophototrophs using input variables described in Supplementary Table S7, and the numerically approximated optimal antenna table. Values of *F*_*P*_ and *F*_*L*_ for the optimal system were recorded at all light intensities, as was the optimal antenna mass, and the ratio of *F*_*p*_ between chlorophototrophs and retinalophototrophs. The light intensity, *F*_*P*_, and *F*_*L*_ of the crossover point at which chlorophototrophy and retinalophototrophy were equivalent was also recorded. See Supplementary Fig. S6 for an analysis of the performance of suboptimal antenna stoichiometries. Units of light intensity in the model are calculated as photons per second per square angstrom, and converted to micromoles of photons per square meter per second for visualization in order to better compare to values in the literature.

## Supplementary information

Supplementary information

## Data Availability

All data used in this work is generated by the included simulation code, which we make available. We include the generated dataset used for this analysis with the available code.

## Code availability

All Python code used in this work is made available upon request and will be posted publicly to the Ratcliff laboratory GitHub (https://github.com/Ratcliff-Lab/Phototrophy-modeling).

## References

[1] Herron, M. D. et al. Cellular differentiation and individuality in the ‘minor’multicellular taxa. *Biol. Rev.* **88**, 844–861 (2013). 23448295 10.1111/brv.12031 PMC4103886

[2] Beukeboom, L. W. & Perrin, N. *The Evolution of Sex Determination* (Oxford University Press, 2014).

[3] Monod, J. On chance and necessity. in *Studies in the Philosophy of Biology: Reduction and Related Problems* 357–375 (Springer, 1974).

[4] De Duve, C. *Singularities: Landmarks on the Pathways of Life* (Cambridge University Press, 2005).

[5] Stroud, J. et al. Priority effects transcend scales and disciplines in biology. *Trends Ecol. Evol.* **39**, 677–688 (2024). 38508922 10.1016/j.tree.2024.02.004

[6] De Meester, L. et al. Evolving perspectives on monopolization and priority effects. *Trends Ecol. Evol.* **31**, 136–146 (2016). 26778169 10.1016/j.tree.2015.12.009

[7] Stroud, J. T. & Ratcliff, W. C. Long-term studies provide unique insights into evolution. *Nature* **639**, 589–601 (2025). 40108318 10.1038/s41586-025-08597-9 PMC12359119

[8] Rolland, J. et al. Conceptual and empirical bridges between micro-and macroevolution. *Nat. Ecol. Evol.* **7**, 1181–1193 (2023). 37429904 10.1038/s41559-023-02116-7

[9] Darwin, C. *The Life and Letters of Charles Darwin: Including an Autobiographical Chapter* Vol. 1 (John Murray, 1887).

[10] Jablonski, D. & Sepkoski, J. J. Jr Paleobiology, community ecology, and scales of ecological pattern. *Ecology* **77**, 1367–1378 (1996). 11539425

[11] Fukami, T. Historical contingency in community assembly: integrating niches, species pools, and priority effects. *Annu. Rev. Ecol., Evol. Syst.* **46**, 1–23 (2015).

[12] Reijenga, B. R., Murrell, D. J. & Pigot, A. L. Priority effects and the macroevolutionary dynamics of biodiversity. *Ecol. Lett.* **24**, 1455–1466 (2021). 33979477 10.1111/ele.13766

[13] Walter, M., Buick, R. & Dunlop, J. Stromatolites 3,400–3,500 Myr old from the North Pole area, Western Australia. *Nature* **284**, 443–445 (1980).

[14] Oliver, T. et al. Time-resolved comparative molecular evolution of oxygenic photosynthesis. *Biochim. Biophys. Acta Bioenerg.* **1862**, 148400 (2021). 33617856 10.1016/j.bbabio.2021.148400 PMC8047818

[15] Cardona, T. A fresh look at the evolution and diversification of photochemical reaction centers. *Photosynth Res.* **126**, 111–134 (2015). 25512103 10.1007/s11120-014-0065-x PMC4582080

[16] Field, C. B. et al. Primary production of the biosphere: integrating terrestrial and oceanic components. *Science* **281**, 237–240 (1998). 9657713 10.1126/science.281.5374.237

[17] Sleep, N. H. & Bird, D. K. Niches of the pre-photosynthetic biosphere and geologic preservation of Earth’s earliest ecology. *Geobiology* **5**, 101–117 (2007).

[18] Bryant, D. A., Hunter, C. N. & Warren, M. J. Biosynthesis of the modified tetrapyrroles-the pigments of life. *J. Biol. Chem.* **295**, 6888–6925 (2020). 32241908 10.1074/jbc.REV120.006194 PMC7242693

[19] Chew, A. G. & Bryant, D. A. Chlorophyll biosynthesis in bacteria: the origins of structural and functional diversity. *Annu. Rev. Microbiol.* **61**, 113–129 (2007). 17506685 10.1146/annurev.micro.61.080706.093242

[20] Nishihara, A. et al. Illuminating the coevolution of photosynthesis and Bacteria. *Proc. Natl. Acad. Sci.* **121**, e2322120121 (2024). 38875151 10.1073/pnas.2322120121 PMC11194577

[21] White, I. S., Canniffe, D. P. & Hitchcock, A. The diversity of physiology and metabolism in chlorophototrophic bacteria. *Adv. Microb. Physiol.* **86**, 1–98 (2025). 40404267 10.1016/bs.ampbs.2025.02.003

[22] Tan, S. et al. Exploring the origins and evolution of oxygenic and anoxygenic photosynthesis in deeply branched cyanobacteriota. *Mol. Biol. Evol.* **41**, msae151 (2024). 39041196 10.1093/molbev/msae151 PMC11304991

[23] Niwa, S. et al. Structure of the LH1–RC complex from Thermochromatium tepidum at 3.0 Å. *Nature* **508**, 228–232 (2014). 24670637 10.1038/nature13197

[24] Noy, D., Moser, C. C. & Dutton, P. L. Design and engineering of photosynthetic light-harvesting and electron transfer using length, time, and energy scales. *Biochim. Biophys. Acta* **1757**, 90–105 (2006). 16457774 10.1016/j.bbabio.2005.11.010

[25] Ward, L. M., Cardona, T. & Holland-Moritz, H. Evolutionary implications of anoxygenic phototrophy in the bacterial phylum Candidatus Eremiobacterota (WPS-2). *Front. Microbiol.* **10**, 1658 (2019). 31396180 10.3389/fmicb.2019.01658 PMC6664022

[26] Xin, Y. et al. Cryo-EM structure of the RC-LH core complex from an early branching photosynthetic prokaryote. *Nat. Commun.* **9**, 1–10 (2018). 29674684 10.1038/s41467-018-03881-x PMC5908803

[27] Fromme, P., Jordan, P. & Krauß, N. Structure of photosystem I. *Biochim. Biophys. Acta Bioenerg.* **1507**, 5–31 (2001). 10.1016/s0005-2728(01)00195-5 11687205

[28] Nagashima, S. & Nagashima, K. V. Comparison of photosynthesis gene clusters retrieved from total genome sequences of purple bacteria. in *Advances in Botanical Research* 151–178 (Elsevier, 2013).

[29] Nawrocki, W. et al. The mechanism of cyclic electron flow. *Biochim. Biophys. Acta Bioenerg*. **1860**, 433–438 (2019). 10.1016/j.bbabio.2018.12.005 30827891

[30] Shikanai, T. Chloroplast NDH: a different enzyme with a structure similar to that of respiratory NADH dehydrogenase. *Biochim. Biophys. Acta* **1857**, 1015–1022 (2016). 26519774 10.1016/j.bbabio.2015.10.013

[31] Larkum, A., Ritchie, R. & Raven, J. Living off the Sun: chlorophylls, bacteriochlorophylls and rhodopsins. *Photosynthetica* **56**, 11–43 (2018).

[32] Bryant, D. A. & Canniffe, D. P. How nature designs light-harvesting antenna systems: design principles and functional realization in chlorophototrophic prokaryotes. *J. Phys. B At. Mol. Opt. Phys.* **51**, 033001 (2018).

[33] Oesterhelt, D. & Stoeckenius, W. Rhodopsin-like protein from the purple membrane of Halobacterium halobium. *Nat. New Biol.* **233**, 149–152 (1971). 4940442 10.1038/newbio233149a0

[34] Béja, O. et al. Bacterial rhodopsin: evidence for a new type of phototrophy in the sea. *Science* **289**, 1902–1906 (2000). 10988064 10.1126/science.289.5486.1902

[35] Rozenberg, A. et al. Microbial rhodopsins: the last two decades. *Annu. Rev. Microbiol.* **75**, 427–447 (2021). 34343014 10.1146/annurev-micro-031721-020452

[36] Sabehi, G. et al. New insights into metabolic properties of marine bacteria encoding proteorhodopsins. *PLoS Biol.* **3**, e273 (2005). 16008504 10.1371/journal.pbio.0030273 PMC1175822

[37] Balashov, S. P. et al. Xanthorhodopsin: a proton pump with a light-harvesting carotenoid antenna. *Science* **309**, 2061–2064 (2005). 16179480 10.1126/science.1118046 PMC3065861

[38] Ernst, O. P. et al. Microbial and animal rhodopsins: structures, functions, and molecular mechanisms. *Chem. Rev.* **114**, 126–163 (2014). 24364740 10.1021/cr4003769 PMC3979449

[39] Palovaara, J. et al. Stimulation of growth by proteorhodopsin phototrophy involves regulation of central metabolic pathways in marine planktonic bacteria. *Proc. Natl. Acad. Sci.* **111**, E3650–E3658 (2014). 25136122 10.1073/pnas.1402617111 PMC4156726

[40] Walter, J. M. et al. Light-powering Escherichia coli with proteorhodopsin. *Proc. Natl. Acad. Sci.* **104**, 2408–2412 (2007). 17277079 10.1073/pnas.0611035104 PMC1892948

[41] Kim, B. H. & Gadd, G. M. *Prokaryotic Metabolism and Physiology* (Cambridge University Press, 2019).

[42] Gómez-Consarnau, L. et al. Microbial rhodopsins are major contributors to the solar energy captured in the sea. *Sci. Adv.* **5**, eaaw8855 (2019). 31457093 10.1126/sciadv.aaw8855 PMC6685716

[43] Cunningham, F. X. et al. Stoichiometry of photosystem I, photosystem II, and phycobilisomes in the red alga Porphyridium cruentum as a function of growth irradiance. *Plant Physiol.* **91**, 1179–1187 (1989). 16667130 10.1104/pp.91.3.1179 PMC1062138

[44] Umena, Y. et al. Crystal structure of oxygen-evolving photosystem II at a resolution of 1.9 Å. *Nature* **473**, 55–60 (2011). 21499260 10.1038/nature09913

[45] Zhang, J. et al. Structure of phycobilisome from the red alga Griffithsia pacifica. *Nature* **551**, 57–63 (2017). 29045394 10.1038/nature24278

[46] Scheuring, S. & Sturgis, J. N. Atomic force microscopy of the bacterial photosynthetic apparatus: plain pictures of an elaborate machinery. *Photosynth. Res.* **102**, 197–211 (2009). 19266309 10.1007/s11120-009-9413-7

[47] Cherezov, V. et al. Room to move: crystallizing membrane proteins in swollen lipidic mesophases. *J. Mol. Biol.* **357**, 1605–1618 (2006). 16490208 10.1016/j.jmb.2006.01.049

[48] Kolber, Z. S. et al. Bacterial photosynthesis in surface waters of the open ocean. *Nature* **407**, 177–179 (2000). 11001053 10.1038/35025044

[49] Lubner, C. E. et al. Solar hydrogen-producing bionanodevice outperforms natural photosynthesis. *Proc. Natl. Acad. Sci.* **108**, 20988–20991 (2011). 22160679 10.1073/pnas.1114660108 PMC3248548

[50] Friedrich, T. et al. Proteorhodopsin is a light-driven proton pump with variable vectoriality. *J. Mol. Biol.* **321**, 821–838 (2002). 12206764 10.1016/s0022-2836(02)00696-4

[51] Lanyi, J. K. Proton transfers in the bacteriorhodopsin photocycle. *Biochim. Biophys. Acta Bioenerg.* **1757**, 1012–1018 (2006). 10.1016/j.bbabio.2005.11.003 16376293

[52] Kirchman, D. L. & Hanson, T. E. Bioenergetics of photoheterotrophic bacteria in the oceans. *Environ. Microbiol. Rep.* **5**, 188–199 (2013). 23584962 10.1111/j.1758-2229.2012.00367.x

[53] Rabbers, I. et al. Metabolism at evolutionary optimal States. *Metabolites* **5**, 311–343 (2015). 26042723 10.3390/metabo5020311 PMC4495375

[54] Flamholz, A. et al. Glycolytic strategy as a tradeoff between energy yield and protein cost. *Proc. Natl. Acad. Sci.* **110**, 10039–10044 (2013). 23630264 10.1073/pnas.1215283110 PMC3683749

[55] Kouyianou, K. et al. The chlorosome of Chlorobaculum tepidum: size, mass and protein composition revealed by electron microscopy, dynamic light scattering and mass spectrometry-driven proteomics. *Proteomics* **11**, 2867–2880 (2011). 21681991 10.1002/pmic.201000494

[56] Mori, M., Marinari, E. & De Martino, A. A yield-cost tradeoff governs Escherichia coli’s decision between fermentation and respiration in carbon-limited growth. *NPJ Syst. Biol. Appl.* **5**, 16 (2019). 31069113 10.1038/s41540-019-0093-4 PMC6494807

[57] Li, Y., Petrov, D. A. & Sherlock, G. Single nucleotide mapping of trait space reveals Pareto fronts that constrain adaptation. *Nat. Ecol. Evol.* **3**, 1539–1551 (2019). 31611676 10.1038/s41559-019-0993-0 PMC7011918

[58] Kopejtka, K. et al. A bacterium from a mountain lake harvests light using both proton-pumping xanthorhodopsins and bacteriochlorophyll-based photosystems. *Proc. Natl. Acad. Sci.* **119**, e2211018119 (2022). 36469764 10.1073/pnas.2211018119 PMC9897461

[59] Hasegawa, M. et al. A unique clade of light-driven proton-pumping rhodopsins evolved in the cyanobacterial lineage. *Sci. Rep.* **10**, 16752 (2020). 33028840 10.1038/s41598-020-73606-y PMC7541481

[60] Choi, A. R. et al. Cyanobacterial light-driven proton pump, Gloeobacter rhodopsin: complementarity between rhodopsin-based energy production and photosynthesis. *PLoS ONE* **9**, e110643 (2014). 25347537 10.1371/journal.pone.0110643 PMC4210194

[61] Marchetti, A. et al. Marine diatom proteorhodopsins and their potential role in coping with low iron availability. *ISME J.* **9**, 2745–2748 (2015). 26023874 10.1038/ismej.2015.74 PMC4817633

[62] Yoshizawa, S. et al. Proton-pumping rhodopsins in marine diatoms. bioRxiv, 10.1101/2022.01.18.476826 (2022).

[63] Yoshizawa, S. et al. Light-driven proton pumps as a potential regulator for carbon fixation in marine diatoms. *Microbes Environ.* **38**, ME23015 (2023). 37344444 10.1264/jsme2.ME23015 PMC10308239

[64] Andrew, S. M. et al. Widespread use of proton-pumping rhodopsin in Antarctic phytoplankton. *Proc. Natl. Acad. Sci.* **120**, e2307638120 (2023). 37722052 10.1073/pnas.2307638120 PMC10523587

[65] Zhang, H. et al. Dinoflagellate proton-pump rhodopsin genes in long island sound: diversity and spatiotemporal distribution. *Microorganisms* **12**, 628 (2024). 38543678 10.3390/microorganisms12030628 PMC10974037

[66] Song, Y. et al. Excitonic structure and charge separation in the heliobacterial reaction center probed by multispectral multidimensional spectroscopy. *Nat. Commun.* **12**, 2801 (2021). 33990569 10.1038/s41467-021-23060-9 PMC8121816

[67] Britt, B. M. For enzymes, bigger is better. *Biophys. Chem.* **69**, 63–70 (1997). 9440209 10.1016/s0301-4622(97)00082-3

[68] Basan, M. et al. Overflow metabolism in Escherichia coli results from efficient proteome allocation. *Nature* **528**, 99–104 (2015). 26632588 10.1038/nature15765 PMC4843128

[69] Scott, M. & Hwa, T. Bacterial growth laws and their applications. *Curr. Opin. Biotechnol.* **22**, 559–565 (2011). 21592775 10.1016/j.copbio.2011.04.014 PMC3152618

[70] Scott, M. et al. Emergence of robust growth laws from optimal regulation of ribosome synthesis. *Mol. Syst. Biol.* **10**, 747 (2014). 25149558 10.15252/msb.20145379 PMC4299513

[71] Connell, J. H. & Slatyer, R. O. Mechanisms of succession in natural communities and their role in community stability and organization. *Am. Nat.* **111**, 1119–1144 (1977).

[72] Blount, Z. D., Borland, C. Z. & Lenski, R. E. Historical contingency and the evolution of a key innovation in an experimental population of Escherichia coli. *Proc. Natl. Acad. Sci.* **105**, 7899–7906 (2008). 18524956 10.1073/pnas.0803151105 PMC2430337

[73] Vannette, R. L. & Fukami, T. Historical contingency in species interactions: towards niche-based predictions. *Ecol. Lett.* **17**, 115–124 (2014). 24341984 10.1111/ele.12204 PMC4344821

[74] Baum, D. A. et al. The ecology–evolution continuum and the origin of life. *J. R. Soc. Interface* **20**, 20230346 (2023). 37907091 10.1098/rsif.2023.0346 PMC10618062

[75] William Schopf, J. The paleobiological record of photosynthesis. *Photosynth. Res.* **107**, 87–101 (2011). 20607406 10.1007/s11120-010-9577-1 PMC3021713

[76] Kaçar, B. Reconstructing early microbial life. *Annu. Rev. Microbiol.* **78**, 463–492 (2024). 39163590 10.1146/annurev-micro-041522-103400

[77] Braun, A. et al. Reviews and syntheses: heterotrophic fixation of inorganic carbon–significant but invisible flux in environmental carbon cycling. *Biogeosciences* **18**, 3689–3700 (2021).

[78] Moody, E. R. et al. The nature of the last universal common ancestor and its impact on the early Earth system. *Nat. Ecol. Evol.* **8**, 1654–1666 (2024). 38997462 10.1038/s41559-024-02461-1 PMC11383801

[79] Sephus, Cathryn D. et al. Earliest photic zone niches probed by ancestral microbial rhodopsins. *Mol. Biol. Evol* **39.5**, msac100 (2022). 35524714 10.1093/molbev/msac100 PMC9117797

[80] DasSarma, S. & Schwieterman, E. W. Early evolution of purple retinal pigments on Earth and implications for exoplanet biosignatures. *Int. J. Astrobiol.* **20**, 241–250 (2021).

[81] Romero, E., Novoderezhkin, V. I. & Van Grondelle, R. Excitation energy transfer and energy conversion in photosynthesis. in *Quantum Effects in Biology* 179–197 (Cambridge University Press, 2014).

[82] Conway Morris, S. *The Runes of Evolution: How the Universe Became Self-Aware* (Templeton Press, 2015).

[83] Stroud, J. T. & Losos, J. B. Ecological opportunity and adaptive radiation. *Annu. Rev. Ecol., Evol. Syst.* **47**, 507–532 (2016).

[84] Miller, A. H., Stroud, J. T. & Losos, J. B. The ecology and evolution of key innovations. *Trends Ecol. Evol.* **38**, 122–131 (2023). 36220711 10.1016/j.tree.2022.09.005

[85] Connolly, J. S., Samuel, E. B. & Janzen, A. F. Effects of solvent on the fluorescence properties of bacteriochlorophyll a. *Photochem. Photobiol.* **36**, 565–574 (1982).

[86] van der Rest, M. & Gingras, G. The pigment complement of the photosynthetic reaction center isolated from Rhodospirillum rubrum. *J. Biol. Chem.* **249**, 6446–6453 (1974). 4214257

[87] Noguchi, T., Hayashi, H. & Tasumi, M. Factors controlling the efficiency of energy transfer from carotenoids to bacteriochlorophyll in purple photosynthetic bacteria. *Biochim. Biophys. Acta Bioenerg.* **1017**, 280–290 (1990).

[88] Yu, L. J. et al. Structural basis for the unusual Qy red-shift and enhanced thermostability of the LH1 complex from Thermochromatium tepidum. *Biochemistry* **55**, 6495–6504 (2016). 27933779 10.1021/acs.biochem.6b00742

[89] Inoue, K. et al. Red-shifting mutation of light-driven sodium-pump rhodopsin. *Nat. Commun.* **10**, 1–11 (2019). 31040285 10.1038/s41467-019-10000-x PMC6491443

[90] Rehorek, M. & Heyn, M. P. Binding of all-trans-retinal to the purple membrane. Evidence for cooperativity and determination of the extinction coefficient. *Biochemistry* **18**, 4977–4983 (1979). 508727 10.1021/bi00589a027

[91] Faizi, M. et al. A model of optimal protein allocation during phototrophic growth. *Biosystems* **166**, 26–36 (2018). 29476802 10.1016/j.biosystems.2018.02.004

[92] She, C. et al. Low-threshold stimulated emission using colloidal quantum wells. *Nano Lett.* **14**, 2772–2777 (2014). 24773282 10.1021/nl500775p

[93] Han, B.-P. A mechanistic model of algal photoinhibition induced by photodamage to photosystem-II. *J. Theor. Biol.* **214**, 519–527 (2002). 11851364 10.1006/jtbi.2001.2468

[94] Kimura, Y. et al. Surface of bacteriorhodopsin revealed by high-resolution electron crystallography. *Nature* **389**, 206–211 (1997). 9296502 10.1038/38323

[95] Tomasello, G., Armenia, I. & Molla, G. The Protein Imager: a full-featured online molecular viewer interface with server-side HQ-rendering capabilities. *Bioinformatics* **36**, 2909–2911 (2020). 31930403 10.1093/bioinformatics/btaa009

