# Supplementary material for: Priority effects inhibit the repeated evolution of phototrophy
Source: Npj Complex. 2026 Feb 2;3(1):9. doi: 10.1038/s44260-026-00069-z (PMC12864036; doi:10.1038/s44260-026-00069-z)
Supplement: Supplementary file 1 — Supplementary information [file 44260_2026_69_MOESM1_ESM.pdf]

Supplement S1

Supplemental Equations & Variable Values

The 'return on investment' of a modern phototrophic system is taken in this work to be the energy flux per unit mass of the dedicated phototrophic machinery at a given light level. Recycling rate of individual proteins within individual modern phototrophic machineries was beyond the scope of this review to incorporate due to the widely varying half-life or dilution rates of different components under different environments, and as such energy fluxes are measured in energy flux per unit mass rather than energy yield per unit of protein synthesis.

To calculate the maximum mass-specific energetic rate of return (V_{max}) on investment of retinalophototrophic versus chlorophototrophic systems, the total mass per functional phototrophic unit (M_{total}), the cycling rate (R_{max}), and the protons pumped per cycle (N_p) must be known. V_{max} is equal to the product of N_p and R_{max} divided by M_{total}, as described in the following equation:

Equation S1: $V_{max} = N_p \cdot R_{max} / M_{total}$

Two retinalophototrophic systems, bacteriorhodopsin and proteorhodopsin, and two chlorophototrophic systems, oxygenic photosynthesizers and purple bacteria, were considered for breadth. Bacteriorhodopsin is described as a functionally monomeric 26 kDa protein [1]and proteorhodopsin is described as a functionally monomeric 27 kDa protein [2].

In the case of complex chlorophototrophic systems, the total mass per functional phototrophic unit must be calculated to include the mass of any antenna complexes associated with a

chlorophototrophic reaction center. This was done by multiplying the mass of each antenna complex by the number of antenna complexes per photosynthetic unit at their in vivo stoichiometry, and adding this to the total mass of the active center. There are few cases in which every reaction center and antenna complex in a single organism are structurally understood and the stoichiometry of each component is known. Thus, in order to estimate the total mass of a functional unit of chlorophototrophic machinery it is necessary to use data from multiple functionally similar organisms to infer an approximate value.

An oxygenic chlorophototrophic reaction center was taken to be a single instance of either photosystem I (PSI) or photosystem II (PSII). As described in Cunningham et al., 1989 [3], the ratio of PSII : PSI : Phycobilisome in the oxygenic red algae *Porphyridium cruentum* (ATCC 50161) in low light (6 μmol photons m^{-2} s^{-1}) is 2.85 : 5.35 : 1 and in high light (280 μmol photons m^{-2} s^{-1}) the ratio is 3.96 : 7.59 : 1. The average across all conditions tested was 3.13 PSII : 6.50 PSI : 1 phycobilisome in this red algae. The mass of PSII is taken to be 350 kDa as described for *Thermosynechococcus vulcanis* in Umena et al., 2011 [4], the mass of PSI is taken to be 356 kDa as described for *Synechococcus elongates* in Fromme et al., 2001 [5], and the mass of a phycobilisome antenna complex is taken to be 16.2 mDa as described for the red algae *Griffithsia pacifica* in Zhang et al., 2017 [6]. This brings the total mass of an approximated stoichiometric unit with 9.63 reaction centers (RCs) and one phycobilisome to approximately 20.2 mDa and the total mass per reaction center to 2098 kDa.

The mass of antenna complexes other than phycobilisomes in oxygenic chlorophototrophs was not considered due to diversity in antenna complexes present in different organisms. The mass of other electron transport chain components or membrane ATPases was not considered due to

likely low but poorly constrained stoichiometries [7], low masses compared to phycobilisomes [8], and their use in multiple cellular processes compared to the comparatively dedicated chlorophototrophic machinery.

Table S1: Mass estimation per oxygenic chlorophototrophic reaction center

Component	Number per stoichiometric unit	Mass
PSII	3.13	350 kDa
PSI	6.50	356 kDa
Phycobilisome	1	16.2 mDa
Total (9.63 RC plus phycobilisome)		20.2 mDa
Mass per RC (M_{total})		2098 kDa

A representative anoxygenic chlorophototrophic system from the purple bacteria species *Rhodospirillum photometricum* was examined for comparison, as described by Scheuring & Sturgis, 2009 [9]. In this species, each type-II reaction center is associated with one complex of 16 LH1 antenna proteins, forming a ‘core complex’, and multiple rings of 9 LH2 antenna proteins. The core complex:LH2 complex ratio was taken to be 3.5, as observed at high light adaptation in this work. The mass of the RCII/LH1 core complex described in Niwa et al., 2014 [10] in the purple bacterium *Thermochromatium tepidum* is 380 kDa. The measured mass of the LH2 complex described by Cherezov et al., 2006 [11] in the purple bacterium *Rps acidophila* is 130 kDa. This brings the total mass of a stoichiometric unit containing a single RC to approximately 835 kDa. Again, the mass of additional electron transport chain components was

not considered due to the multiple roles of these components in other cellular processes and low apparent stoichiometry relative to other chlorophototrophic machinery [7].

Table S2: Mass estimation per anoxygenic purple bacteria chlorophototrophic reaction center

Component	Number per stoichiometric unit	Mass
RC + LH1	1	380 kDa
LH2	3.5	130 kDa
Mass per RC (M_{total})		835 kDa

Field measurements of the maximum cycling rate of chlorophototrophic reaction centers from Kolber et al., 2000 [12] were used along with these figures to estimate the maximum energy flux per unit protein available to chlorophototrophs. In saturating light levels, oxygenic phototrophic phytoplankton were measured to have a maximum sustainable rate of reaction center photocycling of approximately 350 per second, and aerobic anoxygenic phototrophs were measured to reach up to approximately 150 per second. These are broadly consistent with in vitro measurements of photosystem II photocycle rate of more than 200 cycles per second observed in isolated photosystems by Lubner et al., 2011 [13]. Each photocycle of a reaction center was taken to represent two protons pumped across the photosynthetic membrane by the cytochrome b_6f complex in oxygenic chlorophototrophs or other electron transport chain components in anoxygenic chlorophototrophs. Electron transport chains containing the Complex-I like NDH complex (likely capable of pumping additional protons) rather than cytochrome b_6f alone was not considered due to a low apparent rate of cycling and low stoichiometry, suggesting that while necessary for regulatory purposes it is not a primary player in energy metabolism [14].

Microbial rhodopsins were represented by proteorhodopsin which has been measured at being capable of 25 pumping protons per second in Friedrich et al., 2002 [15] and bacteriorhodopsin measured at approximately 50-100 protons per second in Béja et al., 2000 [2] and Lanyi, 2006 [16]. We took the bacteriorhodopsin maximum cycling rate to be 100 protons per second. In all cases, energy flux was calculated as described in equation S1.

Table S3: Maximum energy flux per unit mass for chlorophototrophy and retinalophototrophy

	Proteorhodopsin	Bacteriorhodopsin	Oxygenic RC	Anoxygenic RC
M_{total} (kDa)	27	26	2098	835
R_{max} (cycles s^{-1})	25	100	350	150
N_p (protons $cycle^{-1}$)	1	1	2	2
$R_{max} \cdot N_p$ (Protons s^{-1})	25	100	700	300
V_{max} (Protons kDa^{-1} s^{-1})	0.93	3.85	0.33	0.36

The response of the energy flux per kilodalton of protein mass (F_P) in chlorophototrophs and retinalophototrophs to varying light levels (L) was modeled via simple Michaelis–Menten kinetics, using the following equation:

Equation S2: $F_P = \frac{V_{max} \cdot L}{K_m + L}$

V_{max} was taken to be the previously calculated maximum energy flux rate of a phototrophic system in protons kDa^{-1} s^{-1}. K_m represents the light level at which the energy flux per functional unit reaches its half-maximum. The K_m for both proteorhodopsin and bacteriorhodopsin was taken to be 2700 µmol m^{-2} s^{-1}, as described for proteorhodopsin in Walter et al., 2007 [17]. The

K_m for oxygenic RCs was taken to be 40 µmol m^{-2} s^{-1} and the K_m for an anoxygenic RC was taken to be 191 µmol m^{-2} s^{-1}, as described in Kirchman and Hanson, 2013 [18].

Table S4: K_m, V_{max} for energy flux per unit mass for chlorophototrophy and retinalophototrophy

	Proteorhodopsin	Bacteriorhodopsin	Oxygenic RC	Anoxygenic RC
K_m (µmol m^{-2} s^{-1})	2700	2700	40	191
V_{max} (Protons kDa^{-1} s^{-1})	0.93	3.85	0.33	0.36

The relationship between light intensity and the energy flux per unit protein per unit incident light for each phototrophic system was calculated using equation S3, derived by dividing Equation S2 by light intensity, yielding the following equation.

Equation S3: $F_L = \frac{V_{max}}{K_m + L}$

The flux F_L, in units of protons kDa^{-1} s^{-1} / (µmol m^{-2} s^{-1}), represents the specific energy flux of a unit of protein machinery per unit incident light, and is reduced upon saturation of the machinery with light. It is thus maximized at low light. The form that this efficiency curve takes is similar to that determined from first-principles modeling of anoxygenic chlorophototrophic machinery in Sener et al., 2019 [19].

The maximum yield per unit incident light was calculated by setting [light intensity] equal to zero, at which point the value of Equation S3 is maximized and the largest marginal return per unit incident light is achieved. This value is represented by the following equation:

Equation S4: $Y_{max} = V_{max}/K_m$

This maximum yield represents the highest efficiency available per available light resource, reached at infinitesimal light levels.

Table S5: Maximum yield Y_{max} for chlorophototrophy and retinalophototrophy

	Proteorhodopsin	Bacteriorhodopsin	Oxygenic RC	Anoxygenic RC
Y_{max} (protons kDa^{-1} s^{-1} / (µmol m^{-2} s^{-1}))	$3.43 \cdot 10^{-4}$	$1.43 \cdot 10^{-3}$	$8.34 \cdot 10^{-3}$	$1.88 \cdot 10^{-3}$

It is important to note that a large fraction of the difference in efficiency per unit incident light between chlorophototrophic and retinalophototrophic machinery is due not to the greater quantum yield of chlorophototrophic machinery per unit absorbed photon, but instead is simply due to a much larger absorption cross section per functional unit due to a much larger amount of dedicated light-gathering protein machinery per unit. However, the total absorption cross section per unit infrastructure for chlorophototrophs and retinalophototrophs is quite similar.

The protein infrastructure mass per unit absorption ($\underline{M_{cs}}$) of a phototrophic system measures how efficiently light is captured. It is calculated from the mass per functional unit (M_{total}) and the absorption cross section per functional unit (Cs). The cross-section per functional unit is taken from Kirchman and Hanson, 2013 [18], in which it is approximated for all rhodopsins (2 $Å^2$), an oxygenic phototroph (100 $Å^2$), and an anoxygenic phototroph (50 $Å^2$) based on a compilation of experiments and the light spectrum available in a marine environment. This is represented by the following equation:

Equation S5: $M_{cs} = M_{total}/Cs$

The mass per unit light-absorbing cross section is measured in in units of kDa $Å^{-2}$.

While a large amount of the difference in efficiency per unit incident light is explicable in terms of the difference in total mass per functional unit, the enhanced quantum yield of chlorophototrophic machinery in terms of protons pumped per photon absorbed is an important difference between the two forms of phototrophy. Thus, to account for the greater yield per absorbed photon, the mass per unit cross section is normalized using the following equation:

Equation S6: $NormalizedM_{cs} = M_{cs}/Yield$

This normalized mass per unit cross section accounts for the fact that photons absorbed by chlorophototrophic machinery are used to energize an electron transport chain and are capable of pumping ~2 protons per photon rather than the 1 proton per photon of retinalophototrophic machinery, and are thus twice as efficient in terms of yield per unit incident light.

Table S6: mass per unit cross section calculation for chlorophototrophy and retinalophototrophy

	Proteorhodopsin	Bacteriorhodopsin	Oxygenic RC	Anoxygenic RC
M_{total} (kDa)	27	26	2098	835
Cs ($Å^2$)	2	2	100	50
M_{cs} (kDa $Å^{-2}$)	13.5	13	20.98	16.7

Yield (protons/photon)	1	1	2	2
Yield-Normalized M_{cs} (kDa Å^{-2})	13.5	13	10.49	8.35

In order to mathematically examine the trade-off between efficiency per unit incident light, and efficiency per unit protein infrastructure, the structure of phototrophic machinery was simplified into an eight-parameter model. All phototrophic systems were conceptualized as an invariant catalytic core coupled to a variable quantity of 'antenna' machinery. Antenna increases absorption cross section while not being catalytically active. The catalytic core transduces light energy into biologically available energy (high potential electrons used to run an electron transport chain in the case of chlorophototrophic machinery, and directly pumped protons in the case of retinalophototrophic machinery). Both components are subject to photodegradation.

The antenna mass will be denoted by the variable *x*, in units of kilodaltons. As the total antenna mass per catalytic core will be allowed to vary, the absorption cross section of the antenna will be denoted by the variable a in units of square angstroms per kilodalton.

The catalytic core mass will be denoted by the variable *k*, in units of kilodaltons. Its intrinsic absorption cross section will be denoted by the variable *b*, in units of square angstroms. Its maximum rate of turnover will be denoted by the variable *Vmax*, in terms of cycles per second. Its yield will be denoted by the variable *Y*, in terms of protons pumped per photocycle.

Michaelis-Menten kinetics were used to model the operation of the phototrophic machinery. As such, the K_m of the system (Equation S7) was taken to be the maximum reaction rate of the central engine over the total absorption cross section of the system:

Equation S7: $K_m = \frac{V_{max}}{ax+b}$

The energy flux per unit phototrophic machinery *F* (protons pumped per second) at a given light intensity is denoted by Equation S8, multiplying the standard Michaelis-Menten reaction rate (using light intensity *L* as substrate availability) by the yield per photocycle:

Equation S8: $F = Y \cdot \frac{V_{max} \cdot L}{K_m + L} = \frac{Y \cdot L \cdot V_{max}}{\left(\frac{V_{max}}{ax+b}\right) + L} = \frac{Y \cdot L \cdot V_{max} \cdot (ax+b)}{V_{max} + L \cdot (ax+b)}$

The energy flux per unit protein F_p (protons pumped per kilodalton per second) is denoted by Equation S9, dividing *F* by the total mass of the phototrophic machinery:

Equation S9: $F_P = \frac{F}{k+x} = Y \cdot \frac{V_{max} \cdot L}{K_m + L} * \frac{1}{k+x} = \frac{Y \cdot L \cdot V_{max} \cdot (ax+b)}{(k+x) \cdot (V_{max} + L \cdot (ax+b))}$

The energy flux per unit incident light (protons pumped per kilodalton per second per unit photons per square angstrom per second) is denoted by equation S10, dividing F_p by *L*:

Equation S10: $F_L = Y \cdot \frac{V_{max} \cdot L}{K_m + L} \cdot \frac{1}{k+x} \cdot \frac{1}{L} = \frac{Y \cdot V_{max} \cdot (ax+b)}{(k+x) \cdot (V_{max} + L \cdot (ax+b))}$

Equations 8 through 10 describe the throughput of phototrophic machinery that is fully functional, but light degrades the machinery via photodegradation. To take this into account, methods of Han, 2002[20] and Faizi et al., 2018[21] were used in which photodegradation is proportional to the rate of photon absorption by excited phototrophic machinery. This follows equation S11, in which the rate of photodegradation v_i in fractions of total protein per second is dependent on the photodegradation rate constant *D* (in units of photon^{-1}), absorption cross section (*ax+b*) in square angstroms, light intensity *L* in photons per square angstrom per second, and fraction of the phototrophic machinery excited P^*.

Equation S11: $v_i = D \cdot (ax + b) \cdot L \cdot P^*$

Using a two-state model, the fraction of phototrophic machinery excited is taken to be equal to the ratio of the current reaction rate to the V_{max} resulting in equation S12:

Equation S12: $v_i = D \cdot (ax + b) \cdot L \cdot \frac{L \cdot V_{max} \cdot (ax+b)}{V_{max} + L \cdot (ax+b)} \cdot \frac{1}{V_{max}} = \frac{D \cdot L^2 \cdot (ax+b)^2}{V_{max} + L \cdot (ax+b)}$

The effect of photodegradation of machinery depends on the average turnover rate of the machinery before it is degraded via other means or diluted away by cell division. In steady state, the fraction of phototrophic protein that is functional (P_f) is related to protein turnover rate (R, in s^{-1}) and v_i by equation S13:

Equation S13: $R = R \cdot P_f + v_i \cdot P_f$

Rearranging this equation after substitution with Equation S13 results in Equation S14 for P_f:

Equation S14: $P_f = \frac{R}{R + v_i} = \frac{R}{R + \frac{D \cdot L^2 \cdot (ax+b)^2}{V_{max} + L \cdot (ax+b)}}$

This equation represents the factor by which previously calculated rates and efficiencies must be multiplied in order to determine the true rate of energy transduction, taking into account protein inactivation by light. Thus, we multiply equations 9 and 10 by equation S14 to obtain equations 15 and 16, which will be used for our final analysis:

Equation S15: $F_P = \frac{Y \cdot L \cdot V_{max} \cdot (ax+b)}{(k+x) \cdot (V_{max} + L \cdot (ax+b))} \cdot \frac{R}{R + \frac{D \cdot L^2 \cdot (ax+b)^2}{V_{max} + L \cdot (ax+b)}}$

Equation S16: $F_L = \frac{Y \cdot V_{max} \cdot (ax+b)}{(k+x) \cdot (V_{max} + L \cdot (ax+b))} \cdot \frac{R}{R + \frac{D \cdot L^2 \cdot (ax+b)^2}{V_{max} + L \cdot (ax+b)}}$

Photodegradation and non-photodegradation protein turnover was considered to be the same for chlorophototrophs and retinalophototrophs. The photodegradation rate constant *D* was taken to be $1.6 \cdot 10^{-6}$ $photon^{-1}$ from a Faizi et al. model of optimal phototrophic growth[21]. The non-photodegradation protein turnover rate *R* depends on the dilution timescale of protein in rapidly dividing cells, or the recycling of protein in non-dividing cells. It was taken to be 0.1 hr^{-1}, or 2.78·10-5 s^{-1}, for all analyses. This results in a half-life of protein machinery of approximately 6.93 hours, comparable to the protein dilution timescale of cyanobacteria undergoing exponential growth in bright light[21, 22].

Retinalophototrophy was represented by bacteriorhodopsin, with all numbers as previously described above (table S6, table S7) except for the absorption cross section which was recalculated to take into account differing absorption across the visible spectrum. This was calculated by averaging the absorption spectrum of a rhodopsin [23] across the PAR (Photosynthetically Active Radiation) range of 400 to 700 nanometers, normalized to an extinction coefficient ε of 62700 $M^{-1}cm^{-1}$ at 549 nm[24]. The absorption spectrum was only measured to 424 nm, and all values between this and 400 nm were represented as the same value as 424 nm. This averaged to an extinction coefficient εa across all PAR of 25667 which using Equation S17[25] is translated into an absorption cross section *b* of 0.982 $Å^2$.

Equation S17: $b = \varepsilon a \cdot \frac{2303}{6.02 \cdot 10^{23} M^{-1} \cdot 1cm}$

Chlorophototrophy was similarly represented by an anoxygenic proteobacterial type 2 RC as described above, since it was the chlorophototrophic system most easily decomposed into a discrete central engine and antenna. The anoxygenic RC engine requires an absorption cross-section b and mass k in isolation without any attached antenna complexes. Absorption cross

section was approximated by multiplying the number of bound photopigments by their cross sections. The composition of the *Thermochromatium tepidum* reaction center was used to extrapolate the absorption cross section of a minimal chlorophototrophic reaction center. This contains four bacteriochlorophyll and two bacteriopheophytin molecules and one integral spirilloxanthin carotenoid[10, 26, 27]. Absorption spectra were obtained via personal communication with Dr. Canniffe of the University of Liverpool. The spectrum of bacteriochlorophyll was normalized to an extinction coefficient of 92000 $M^{-1}cm^{-1}$ at 781 nm[28], that of bacteriopheophytin a to 45100 $M^{-1}cm^{-1}$ at 747 nm[29], and that of spirilloxanthin to 101400 $M^{-1}cm^{-1}$ at 525 nm[29]. All were averaged across the PAR range of 400 to 700 nm. Using equation S17, the absorption cross section of bacteriochlorophyll came to 0.476 $Å^2$, bacteriopheophytin came to 0.236 $Å^2$, and spirilloxanthin came to 1.390 $Å^2$. The spirilloxanthin carotenoid was considered to transfer 40% of its absorbed photons, as it has a role in quenching and a low quantum efficiency[30]. Thus, the total absorption cross section of the *T tepidum* central reaction center comes to approximately 2.932 $Å^2$. Mass was approximated by subtracting the mass of the LH1 ring, leaving a central engine with a core homologous to those of other reaction centers with a mass of approximately 150 kDa.

Lastly, an absorption cross section per unit mass of antenna was required. The same value was used for both chlorophototrophic antennas, and hypothetical antennas connected to rhodopsins. This was taken to be approximately 0.163 $Å^2$ kDa^{-1}. This is the value obtained by dividing the sum of the absorption cross section of all pigments in the LH2 antenna complex[11] (18 bacteriochlorophyll and 9 spirilloxanthin) by the mass of the LH2 antenna complex (130 kDa). The full absorption cross section for spirilloxanthin was used, as the quantum efficiency of

photon absorption by antenna carotenoids in LH1 and LH2 can approach 100%[30]. All variables are recorded in Table S7.

Table S7: Variables for calculation of efficiency per unit light and efficiency per unit infrastructure of chlorophototrophic and retinalophototrophic machinery

	Bacteriorhodopsin	Anoxygenic RC
k (kDa)	26	150
b (Å^2)	0.982	2.392
Vmax (s^{-1})	100	150
Y (protons cycle^{-1})	1	2
a (Å^2 kDa^{-1})	0.163	0.163
D (photon^{-1})	$1.6 \cdot 10^{-6}$	$1.6 \cdot 10^{-6}$
R (s^{-1})	$2.78 \cdot 10^{-5}$	$2.78 \cdot 10^{-5}$

Supplemental Results

Supplemental figure 2A, and 2C through 2F depicts the emergent trade-off between efficiency per unit protein and efficiency per unit incident light for chlorophototrophs and retinalophototrophs in the form of a plot of the optimal F_P versus F_t. Every separate point on the solid green and purple lines represents the efficiencies of a different optimal phototrophic system at a given light intensity. At all other light intensities they are strictly inferior in all respects to a better-optimized phototrophic system of the same type, except in the degenerate case of a system which is best with zero antenna at a range of light intensities, which occurs at the highest light intensities for chlorophototrophy. The optimal systems together define a Pareto front, representing a trade-off between efficiency per unit protein infrastructure and efficiency per unit

light resource. At high light levels (above ~185 micromoles of photons per square meter per square meter per second) retinalophototrophs dominate with high energy flux per unit protein, while at low light levels retinalophototrophs dominate with high efficiency per unit incident light.

Notably, as the equations for FP and F_L differ only by a factor of light intensity L, regardless of the system being used, Equation S18 holds:

Equation S18: $\frac{F_L}{F_P} = \frac{1}{L}$

This means that at a given light intensity, any phototrophic system regardless of its properties will lie somewhere on a line passing through the origin with a slope inversely proportional to the light intensity.

The curves describing the optimization of the two systems cross at a critical light intensity of 186 micromoles of photons per square meter per second, with retinalophototrophy producing both higher optimal F_P and F_L at higher light intensities but chlorophototrophy superior at lower light intensities. Again, as the equations for F_P and F_L are identical except for a factor of light intensity, at a given light intensity the advantage of a given phototrophic system is identical for both.

Supplemental figure 2B illustrates the optimal antenna mass for chlorophototrophs and retinalophototrophs at different light intensities. At the crossover point, the optimal chlorophototroph is calculated to have an antenna mass of 95.4 kDa and the optimal retinalophototroph is calculated to have an antenna mass of 60.8 kDa. These values climb without bound as light level decreases to zero, but as light intensity increases they reach zero for

chlorophototrophy by 1845 micromoles of photons per kilodalton per second, and decrease to only 2.1 kDa for retinalophototrophy at the maximum light intensity examined.

These values comport well with the observed makeup of real-world phototrophic machinery. Chlorophototrophs' antenna complexes range all the way in size up to massive crystalline chlorosomes which can be significant fractions of the size of a bacterium[31] and have even been observed on the ocean floor[32]. There is no known chlorophototrophic reaction center in nature that does not bear antenna complexes, with all type I reaction centers bearing "core" antennas which are part of the same polypeptide chain, and type II reaction centers either bearing a LH1 ring permanently associated with the complex in the case of anoxygenic reaction centers or a core antenna related to that of type I reaction centers but in a separate polypeptide in the case of photosystem II. The minimum antenna complex associated with a reaction center is approximately 130 kDa in the form of the sum of the core antennas of the dimeric heliobacteria reaction center[33] – remarkably close to the predicted value of the smallest antenna.

Real-world retinalophototrophic machinery has only ever been observed to contain single carotenoid molecules attached to the sides of xanthorhodopsin molecules as antennas[34], rather than any more elaborate antenna complexes. The calculated largest optimal antenna of the retinalophototrophic machinery closest to the crossover point is approximately the same size as the smallest known antenna complex – a single copy of the heliobacterial core antenna at 65 kDa. This would, however, only be useful at the very lowest light levels that retinalophototrophs are superior at, possibly limiting the utility of tying these sorts of antenna complexes into this pathway.

Supplemental figure 3 illustrates the relative advantage of chlorophototrophs and retinalophototrophs at different light intensities. This was calculated by computing the ratio of F_P for the two systems at all light intensities. At low light intensities chlorophototrophy has nearly a 2-fold advantage in our model, while at high light intensities the advantage shifts to retinalophototrophy and increases to over 3-fold in unfiltered direct sunlight.

Importantly, the crossover point between the chlorophototroph and retinalophototrophic curve is dependent on the turnover rate R of the phototrophic machinery – as turnover rate decreases, there is more opportunity for photodegradation and small differences in photodegradation rate compound into larger differences in the functional fraction of machinery. This variable is highly dependent on metabolic state and growth rate, and so any one value is only representative of a small set of physiological conditions. We performed an analysis of the sensitivity of the modeled crossover light level to the base protein turnover rate R. See Supplemental figure 4 for a plot of crossover light intensity versus both protein turnover rate R, and the half-life of phototrophic protein machinery. Half-life was calculated using Equation S19.

Equation S19: $Half\ Life = \frac{\ln(2)}{R}$

At extremely fast physiologically unlikely turnover rates, the crossover point rises rapidly in light intensity, with the crossover point reaching 578 micromoles of photons per square meter per second at a R of 1 hr^{-1} and a half-life of 0.69 hours, faster than any known phototrophic doubling time. As turnover rates slow down the crossover point drops in light intensity, with it reaching 47 micromoles of photons per square meter per second at a half-life/doubling time of 100 hours and as low as 17.7 micromoles of photos per square meter per second at an R of 0.001 hr^{-1} and a half-life of 693 hours. As turnover rate can depend on either protein recycling or

dilution due to cell division, we should expect a crossover between chlorophototrophic and retinalophototrophic advantage at lower light levels when considering slow-growing metabolically quiescent cells as compared to fast-growing metabolically active cells, consistent with its observed role in preventing starvation[35]. While these crossover light levels vary over a factor of thirty as we allow the recycling and dilution rate R to vary 0.001 hr^{-1} to 1 hr^{-1}, they remain much dimmer than direct sunlight and the trade-off curves of F_L vs F_P retain a similar qualitative relationship – see supplemental figure 4 C through E.

In order to confirm the real-world ecological significance of the calculated critical light intensity, we analyzed the distribution of retinal and chlorophylls at depth in the ocean as previously described by Gómez-Consarnau et al[36]. Taking raw data from a transect of the Mediterranean Sea, we isolated every datapoint and examined the total flux of photosynthetically active radiation (PAR), the concentration of retinal pigment, and the total quantity of chlorophyll-a plus the concentration of bacteriochlorophyll. Unmeasurably low values were taken to be zero. We sorted these datapoints by PAR intensity, performed a rolling average of 10 datapoints to smooth noise, and normalized pigment concentrations to their average concentration across all datapoints to account for different average absolute concentrations of chlorophyll and retinal pigments. Supplemental figure 5 illustrates the resultant summary of relative concentration of chlorophototrophic and retinalophototrophic pigments at different PAR levels in a transect of the open ocean. The pattern is roughly consistent with the relative advantages of chlorophototrophy versus retinalophototrophy as calculated in our model – chlorophototrophic pigments are above their average concentration at light intensities below our calculated crossover point, and retinalophototrophic pigments are above their average concentration at light intensities above our calculated crossover point, although the threshold is not sharp. This could be the result of the

previously noted dependence of the favorability of phototrophic mode on the recycling rate of the machinery, and the presence of many different growth rate populations in the ocean. This may also be because the ocean is dynamic and mixed rather than perfectly stratified by light level, and because even as both chlorophototrophy and retinalophototrophy produce biological energy, they do have important physiological and ecological differences – for example retinalophototrophy cannot produce biomass no matter what light intensity it is exposed to, and requires no limiting iron in resource-poor environments. We thus observe qualitative evidence for the accuracy of our calculation of the ecological partitioning of phototrophic niche space between chlorophototrophs and retinalophototrophs.

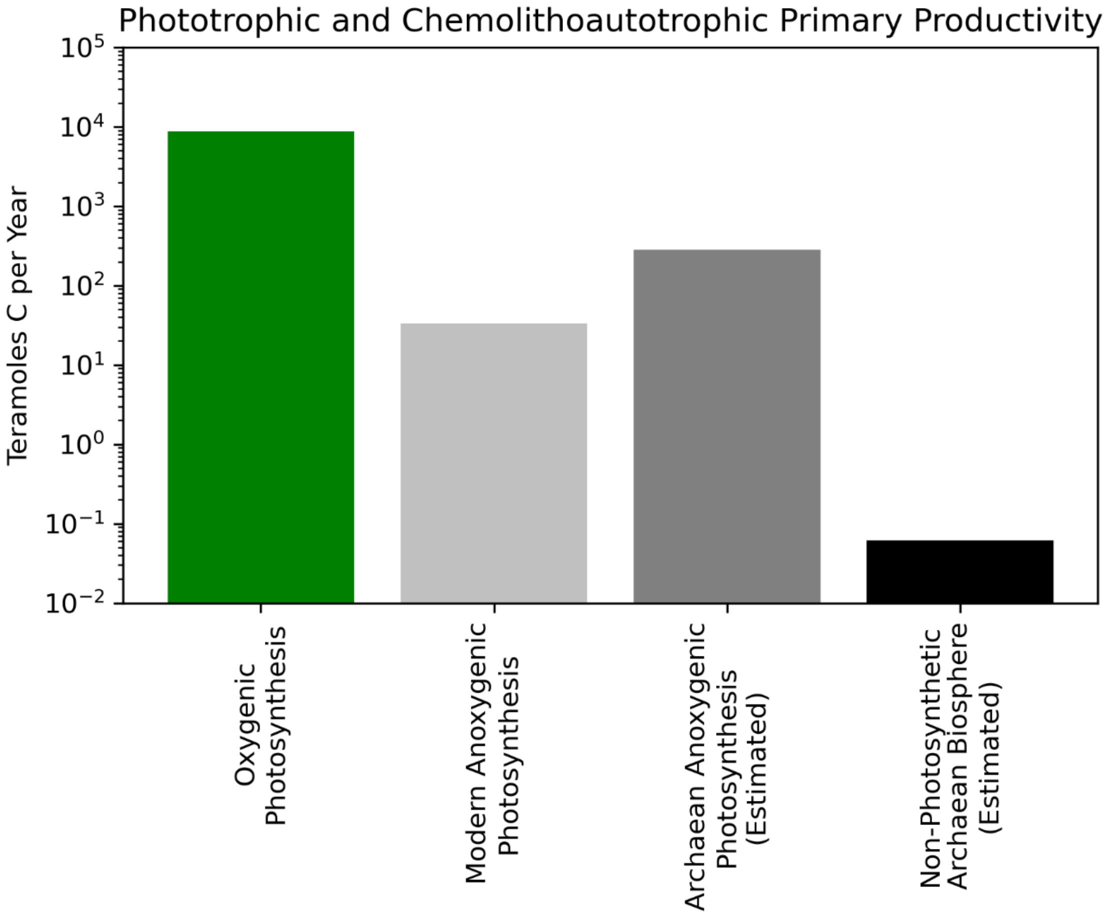

Supplemental Figure S1: Phototrophic vs Chemolithoautotrophic Primary Production

Comparison between carbon flux through biospheres driven by photosynthesis and chemolithoautotrophy. Modern oxygenic photosynthesis drives carbon fixation of nearly 9,000 teramoles per year[37]. While modern anoxygenic photosynthesis represents at most ~2.7 teramoles per year [38], estimates of anoxygenic photosynthesis in the Archaean are ~280 teramoles per year [39]. Estimates of possible carbon flux through a pre-photosynthetic, entirely chemolithotrophic biosphere driven by Archaean geochemical fluxes alone are circa 0.06 teramoles per year [40].

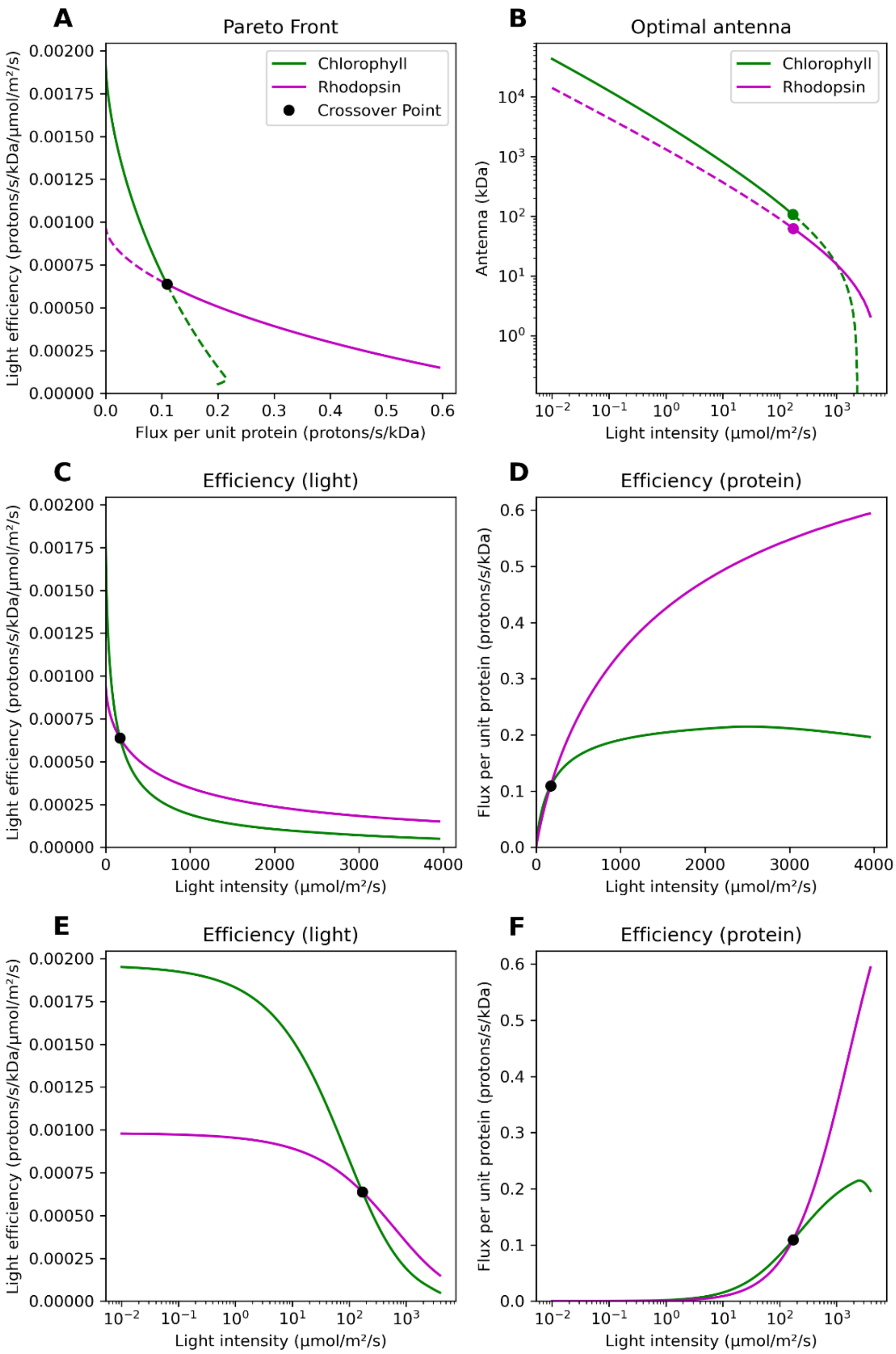

Supplemental Figure S2: Model of Optimal Chlorophototrophic and Retinalophototrophic Machinery

A) Modeled trade-off between efficiency per unit protein and efficiency per unit light for

chlorophototrophy and retinalophototrophy. The optimal system at a given light intensity is pictured as a solid line, with the sub-optimal system at a given light intensity pictured as a dotted line. The crossover point is indicated with an open circle. B) The optimal antenna mass modeled for chlorophototrophs and retinalophototrophs graphed across different light intensities, pictured as in the previous panel. The optimal antenna for both systems rises to arbitrarily large masses at low light levels, before falling to zero at less than the maximum modeled light intensity for chlorophototrophs and near zero at the maximum modeled light intensity for retinalophototrophs. The crossover point at which both systems are modeled as equivalent is indicated via solid circles. C) Efficiency per unit light versus light intensity, with light intensity on a linear scale. Crossover point indicated. D) Energy flux per unit protein, with light intensity on a linear scale. Crossover point is indicated. E) Efficiency per unit light versus light intensity, with light intensity on a logarithmic scale. F) Energy flux per unit protein versus light intensity, with light intensity on a logarithmic scale.

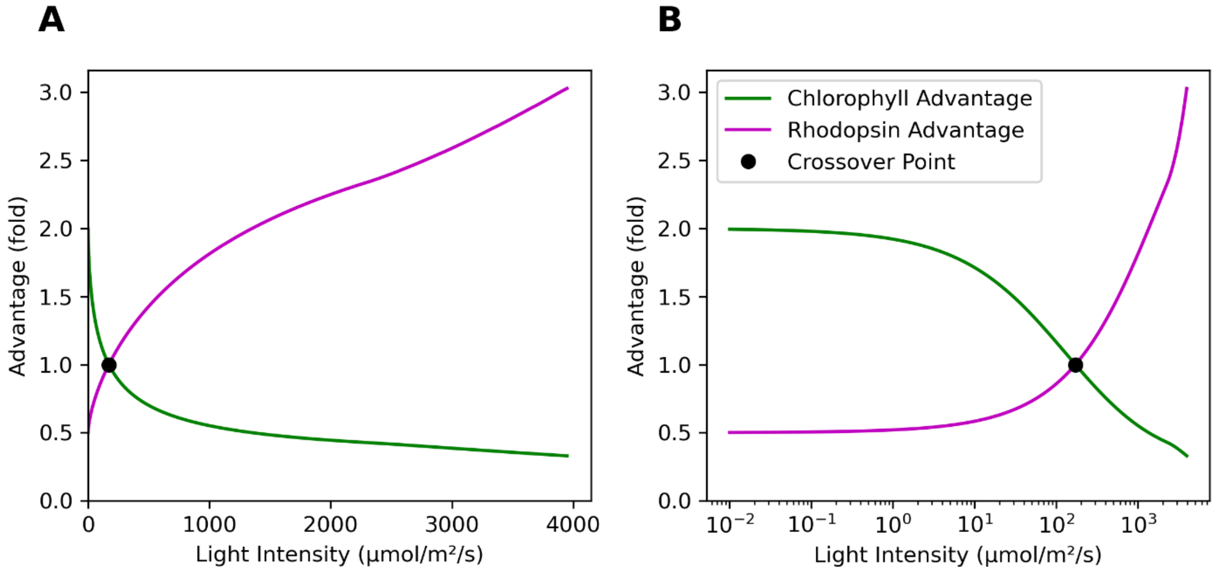

Supplemental Figure S3: Relative Advantages of Chlorophototrophs and Retinalophototrophs at Different Light Intensities

A) The fold advantage of the optimal calculated chlorophototrophic system and retinalophototrophic system at different light intensities. Both systems are evenly matched in terms of energy flux per unit protein, or efficiency per unit light, at a light intensity of 186 micromoles of photons per square meter per second. At low light intensity chlorophototrophs approach a nearly 2-fold advantage, while at high light levels the advantage of retinalophototrophs exceeds 3-fold. B) The same data, on a logarithmic light scale to enhance legibility.

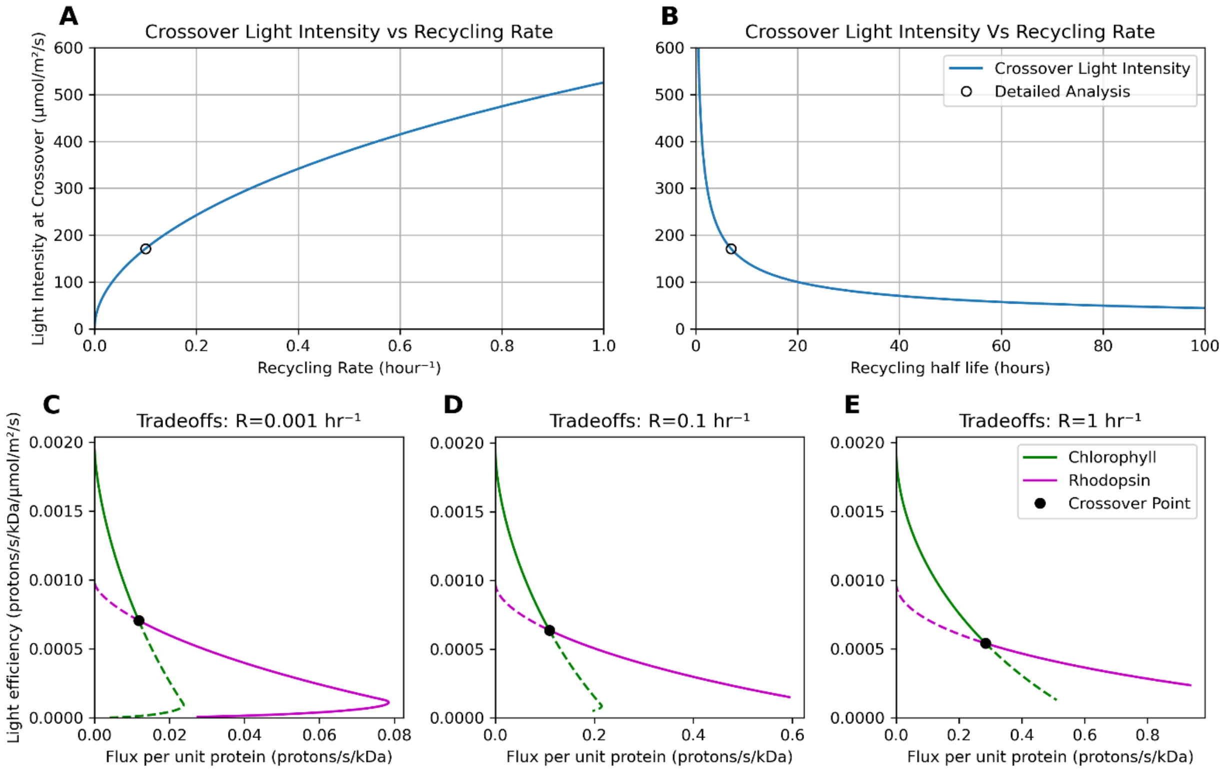

Supplemental Figure S4: Sensitivity of Crossover Value to Degradation Rate Constant

A) Sensitivity of the modeled crossover point of chlorophototrophy and retinalophototrophy to the degradation rate constant R. Open circle indicates the value used for detailed analysis in this publication. At a degradation constant of 0.001 hr^{-1} (half-life of 693 hours) a crossover point between chlorophototrophy and retinalophototrophy occurs at 17.7 μmols m^{-2}s^{-1}, at 0.1 hr^{-1} (half-life of 6.9 hours) it occurs at 185.6 μmols m^{-2}s^{-1}, and at 1 hr^{-1} (half-life of 0.69 hours) it occurs at 577.7 μmols m^{-2}s^{-1}. B) Sensitivity of the modeled crossover point to the non-photodegradation half-life of phototrophic machinery. Open circle indicates the value used for detailed analysis in this publication. C) Trade-off of efficiency per unit protein versus efficiency per incident light with recycling/dilution rate of 0.001 hr^{-1}. D) Trade-off of efficiency per unit protein versus efficiency per incident light with recycling/dilution rate of 0.1 hr^{-1}. E) Trade-off of efficiency per unit protein versus efficiency per incident light with recycling/dilution rate of 1 hr^{-1}. Trade-offs remain similar in form.

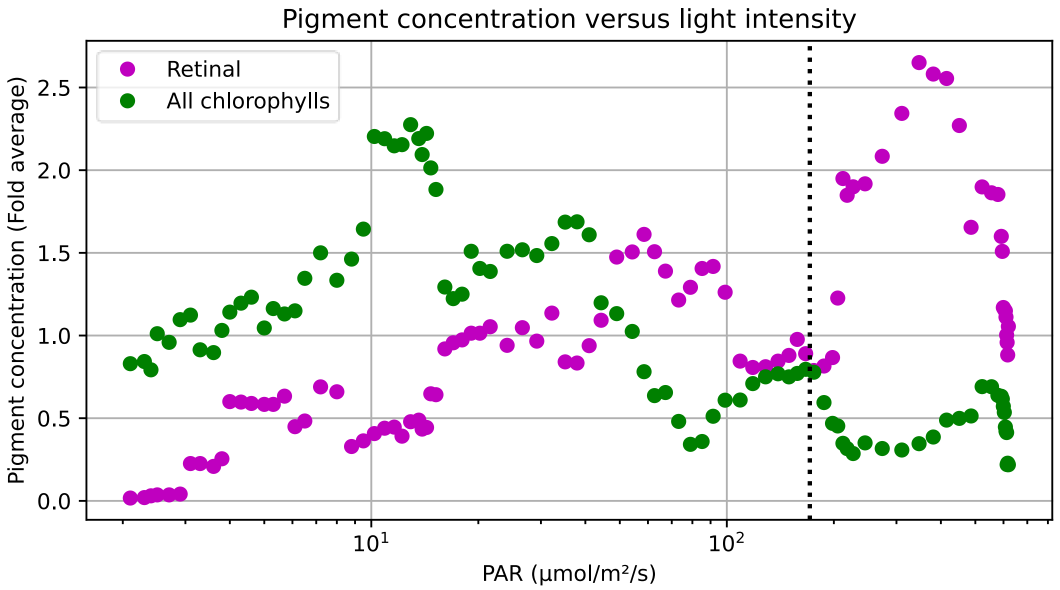

Supplemental Figure S5: Comparison of Calculated Properties of Chlorophototrophs and Retinalophototrophs, and Their Actual Distribution (Gómez-Consarnau et al.)

Smoothed data from Gómez-Consarnau et al.[36] on the distribution of chlorophototrophic and retinalophototrophic pigments in the water column according to photosynthetically active radiation (PAR). All values are normalized to the average concentration of pigment across the whole dataset. Dotted line indicates the modeled theoretical crossover point at which chlorophototrophy and retinalophototrophy are equivalent. The highest retinal pigment concentrations are observed above the modeled crossover point, and the highest chlorophototrophic pigment levels are observed below the crossover point.

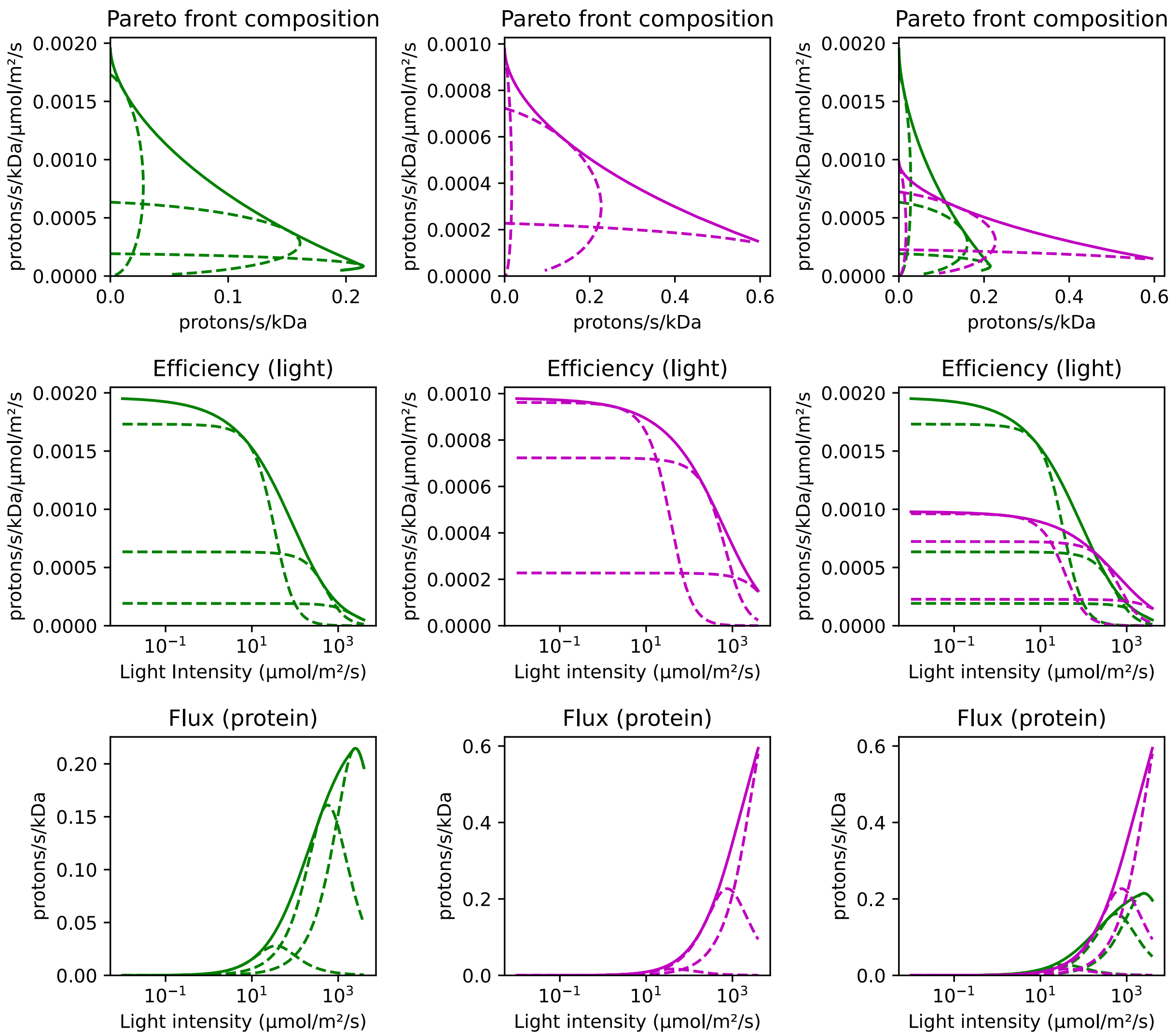

Supplemental Figure S6: Individual Realizations of Phototrophic Machinery Produce the Pareto Front of Optimal Machineries At All Light Intensities

Comparison of individual phototrophic machineries versus the pareto front of all optimal machineries. Solid lines indicate the pareto front (top row), efficiency per unit light (middle row), and flux per unit protein (bottom row) of optimal chlorophototrophs (solid green line) and optimal retinalophototrophs (solid purple line). Individual realizations with different quantities of antenna complex (0, 50, and 1000 kDa) are indicated by dotted lines. Each has a light intensity at which it maximizes flux per unit protein, and all performance curves touch the optimal curve at one point.

References

1. Oesterhelt, D. and W. Stoeckenius, *Rhodopsin-like protein from the purple membrane of Halobacterium halobium.* Nature new biology, 1971. **233**(39): p. 149-152.
2. Béja, O., et al., *Bacterial rhodopsin: evidence for a new type of phototrophy in the sea.* Science, 2000. **289**(5486): p. 1902-1906.
3. Cunningham, F.X., et al., *Stoichiometry of photosystem I, photosystem II, and phycobilisomes in the red alga Porphyridium cruentum as a function of growth irradiance.* Plant physiology, 1989. **91**(3): p. 1179-1187.
4. Umena, Y., et al., *Crystal structure of oxygen-evolving photosystem II at a resolution of 1.9 Å.* Nature, 2011. **473**(7345): p. 55-60.
5. Fromme, P., P. Jordan, and N. Krauß, *Structure of photosystem I.* Biochimica et Biophysica Acta (BBA)-Bioenergetics, 2001. **1507**(1-3): p. 5-31.
6. Zhang, J., et al., *Structure of phycobilisome from the red alga Griffithsia pacifica.* Nature, 2017. **551**(7678): p. 57-63.
7. Singharoy, A., et al., *Atoms to Phenotypes: Molecular Design Principles of Cellular Energy Metabolism.* Cell, 2019. **179**(5): p. 1098-1111. e23.
8. Muench, S.P., J. Trinick, and M.A. Harrison, *Structural divergence of the rotary ATPases.* Quarterly reviews of biophysics, 2011. **44**(3): p. 311-356.
9. Scheuring, S. and J.N. Sturgis, *Atomic force microscopy of the bacterial photosynthetic apparatus: plain pictures of an elaborate machinery.* Photosynthesis research, 2009. **102**(2-3): p. 197-211.
10. Niwa, S., et al., *Structure of the LH1–RC complex from Thermochromatium tepidum at 3.0 Å.* Nature, 2014. **508**(7495): p. 228-232.
11. Cherezov, V., et al., *Room to move: crystallizing membrane proteins in swollen lipidic mesophases.* Journal of molecular biology, 2006. **357**(5): p. 1605-1618.
12. Kolber, Z.S., et al., *Bacterial photosynthesis in surface waters of the open ocean.* Nature, 2000. **407**(6801): p. 177-179.
13. Lubner, C.E., et al., *Solar hydrogen-producing bionanodevice outperforms natural photosynthesis.* Proceedings of the National Academy of Sciences, 2011. **108**(52): p. 20988-20991.
14. Nawrocki, W., et al., *The mechanism of cyclic electron flow.* Biochimica et Biophysica Acta (BBA)-Bioenergetics, 2019.
15. Friedrich, T., et al., *Proteorhodopsin is a light-driven proton pump with variable vectoriality.* Journal of molecular biology, 2002. **321**(5): p. 821-838.
16. Lanyi, J.K., *Proton transfers in the bacteriorhodopsin photocycle.* Biochimica et Biophysica Acta (BBA)-Bioenergetics, 2006. **1757**(8): p. 1012-1018.
17. Walter, J.M., et al., *Light-powering Escherichia coli with proteorhodopsin.* Proceedings of the National Academy of Sciences, 2007. **104**(7): p. 2408-2412.
18. Kirchman, D.L. and T.E. Hanson, *Bioenergetics of photoheterotrophic bacteria in the oceans.* Environmental microbiology reports, 2013. **5**(2): p. 188-199.
19. Sener, M., et al., *Overall energy conversion efficiency of a photosynthetic vesicle.* elife, 2016. **5**: p. e09541.
20. Han, B.-P., *A mechanistic model of algal photoinhibition induced by photodamage to photosystem-II.* Journal of theoretical biology, 2002. **214**(4): p. 519-527.
21. Faizi, M., et al., *A model of optimal protein allocation during phototrophic growth.* Biosystems, 2018. **166**: p. 26-36.

22. Zavřel, T., et al., *Characterization of a model cyanobacterium Synechocystis sp. PCC 6803 autotrophic growth in a flat-panel photobioreactor.* Engineering in Life Sciences, 2015. **15**(1): p. 122-132.
23. Inoue, K., et al., *Red-shifting mutation of light-driven sodium-pump rhodopsin.* Nature communications, 2019. **10**(1): p. 1-11.
24. Rehorek, M. and M.P. Heyn, *Binding of all-trans-retinal to the purple membrane. Evidence for cooperativity and determination of the extinction coefficient.* Biochemistry, 1979. **18**(22): p. 4977-4983.
25. She, C., et al., *Low-threshold stimulated emission using colloidal quantum wells.* Nano letters, 2014. **14**(5): p. 2772-2777.
26. Yu, L.J., et al., *Structural Basis for the Unusual Qy Red-Shift and Enhanced Thermostability of the LH1 Complex from Thermochromatium tepidum.* Biochemistry, 2016. **55**(47): p. 6495-6504.
27. Noy, D., C.C. Moser, and P.L. Dutton, *Design and engineering of photosynthetic light-harvesting and electron transfer using length, time, and energy scales.* Biochim Biophys Acta, 2006. **1757**(2): p. 90-105.
28. Connolly, J.S., E.B. Samuel, and A.F. Janzen, *Effects of solvent on the fluorescence properties of bacteriochlorophyll a.* Photochemistry and photobiology, 1982. **36**(5): p. 565-574.
29. van der Rest, M. and G. Gingras, *The pigment complement of the photosynthetic reaction center isolated from Rhodospirillum rubrum.* Journal of Biological Chemistry, 1974. **249**(20): p. 6446-6453.
30. Noguchi, T., H. Hayashi, and M. Tasumi, *Factors controlling the efficiency of energy transfer from carotenoids to bacteriochlorophyll in purple photosynthetic bacteria.* Biochimica et Biophysica Acta (BBA)-Bioenergetics, 1990. **1017**(3): p. 280-290.
31. Kouyianou, K., et al., *The chlorosome of Chlorobaculum tepidum: size, mass and protein composition revealed by electron microscopy, dynamic light scattering and mass spectrometry-driven proteomics.* Proteomics, 2011. **11**(14): p. 2867-80.
32. Beatty, J.T., et al., *An obligately photosynthetic bacterial anaerobe from a deep-sea hydrothermal vent.* Proceedings of the National Academy of Sciences, 2005. **102**(26): p. 9306-9310.
33. Gisriel, C., et al., *Structure of a symmetric photosynthetic reaction center–photosystem.* Science, 2017. **357**(6355): p. 1021-1025.
34. Balashov, S.P., et al., *Xanthorhodopsin: a proton pump with a light-harvesting carotenoid antenna.* Science, 2005. **309**(5743): p. 2061-4.
35. Gómez-Consarnau, L., et al., *Proteorhodopsin Phototrophy Promotes Survival of Marine Bacteria during Starvation.* PLOS Biology, 2010. **8**(4): p. e1000358.
36. Gómez-Consarnau, L., et al., *Microbial rhodopsins are major contributors to the solar energy captured in the sea.* Science advances, 2019. **5**(8): p. eaaw8855.
37. Field, C.B., et al., *Primary production of the biosphere: integrating terrestrial and oceanic components.* science, 1998. **281**(5374): p. 237-240.
38. Raven, J.A., *Contributions of anoxygenic and oxygenic phototrophy and chemolithotrophy to carbon and oxygen fluxes in aquatic environments.* Aquatic Microbial Ecology, 2009. **56**(2-3): p. 177-192.
39. Canfield, D.E., M.T. Rosing, and C. Bjerrum, *Early anaerobic metabolisms.* Philosophical Transactions of the Royal Society B: Biological Sciences, 2006. **361**(1474): p. 1819-1836.
40. Sleep, N.H. and D.K. Bird, *Niches of the pre-photosynthetic biosphere and geologic preservation of Earth's earliest ecology.* Geobiology, 2007. **5**(2): p. 101-117.
